# Mitochondrial Protein Lipoylation and the 2-Oxoglutarate Dehydrogenase Complex Controls HIF1α Stability in Aerobic Conditions

**DOI:** 10.1016/j.cmet.2016.09.015

**Published:** 2016-11-08

**Authors:** Stephen P. Burr, Ana S.H. Costa, Guinevere L. Grice, Richard T. Timms, Ian T. Lobb, Peter Freisinger, Roger B. Dodd, Gordon Dougan, Paul J. Lehner, Christian Frezza, James A. Nathan

**Affiliations:** 1 Department of Medicine, Cambridge Institute for Medical Research, University of Cambridge, Cambridge, CB2 0XY, UK; 2 Hutchinson MRC Cancer Unit, University of Cambridge, Cambridge, CB2 0XZ, UK; 3 Kreiskliniken Reutlingen GmbH, 72764 Stuttgart, Germany; 4 Department of Medicine, University of Cambridge, Cambridge, CB2 0XY, UK; 5 Wellcome Trust Sanger Institute, Wellcome Trust Genome Campus, Cambridge, CB10 1SA, UK

## Abstract

Hypoxia-inducible transcription factors (HIFs) control adaptation to low oxygen environments by activating genes involved in metabolism, angiogenesis, and redox homeostasis. The finding that HIFs are also regulated by small molecule metabolites highlights the need to understand the complexity of their cellular regulation. Here we use a forward genetic screen in near-haploid human cells to identify genes that stabilize HIFs under aerobic conditions. We identify two mitochondrial genes, oxoglutarate dehydrogenase (OGDH) and lipoic acid synthase (LIAS), which when mutated stabilize HIF1α in a non-hydroxylated form. Disruption of OGDH complex activity in OGDH or LIAS mutants promotes L-2-hydroxyglutarate formation, which inhibits the activity of the HIFα prolyl hydroxylases (PHDs) and TET 2-oxoglutarate dependent dioxygenases. We also find that PHD activity is decreased in patients with homozygous germline mutations in lipoic acid synthesis, leading to HIF1 activation. Thus, mutations affecting OGDHC activity may have broad implications for epigenetic regulation and tumorigenesis.

## Highlights

• A forward genetic screen identifies OGDH and LIAS as novel regulators of HIF1
• Disruption of OGDH and LIAS results in 2-OG accumulation and production of L-2-HG
• L-2-HG stabilizes HIF1α in aerobic conditions by inhibiting prolyl-hydroxylation
• Human germline mutations in lipoic acid synthesis activate HIF1 due to L-2-HG accumulation

## Graphical Abstract

**Figure:**
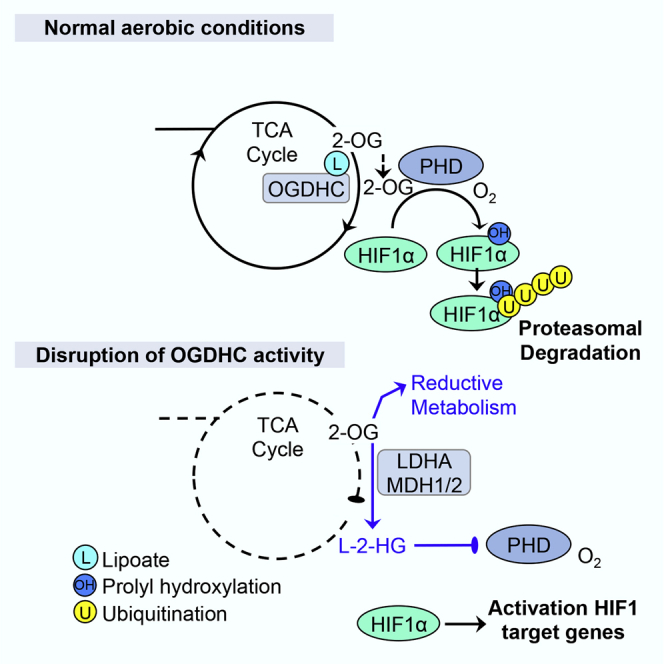

## Teaser

Burr et al. identify using an unbiased forward genetic screen that disrupting the oxoglutarate dehydrogenase complex or mitochondrial lipoylation stabilizes HIF1α through inhibition of prolyl-hydroxylation by L-2-HG. They also show that human germline mutations in lipoic acid synthesis genes show HIF1 activation through the same L-2-HG-dependent mechanism.

## Introduction

Hypoxia-inducible transcription factors (HIFs) are central to the metazoan hypoxia response and are key mediators of glycolysis, regulating approximately 600 genes to promote cell survival in low oxygen tensions (Benita et al., 2009, Manalo et al., 2005). Undesirable consequences of their activation can promote tumor formation, lead to the development of pulmonary hypertension, and result in altered immune responses (Kaelin, 2008, Maxwell and Ratcliffe, 2002). Therefore, understanding how HIFs are regulated is of fundamental biological importance.

The principal mechanism for regulating HIFs relies on constitutive hydroxylation and ubiquitin-mediated proteasomal degradation of the HIFα subunit in aerobic conditions, which prevents the formation of an active heterodimeric transcription factor with HIFβ (aryl hydrocarbon receptor nuclear translocator [ARNT]). Prolyl hydroxylase domain enzymes (PHDs) act as oxygen sensors within cells and hydroxylate HIFα at two conserved proline residues within its oxygen-dependent degradation domain (ODD) (Bruick and McKnight, 2001, Epstein et al., 2001). This hydroxylation signals the recruitment of the Von Hippel (pVHL) ubiquitin E3 ligase complex (Maxwell et al., 1999) and the subsequent proteasomal degradation of HIFα. As PHDs are members of the 2-oxoglutarate- (2-OG, or α-ketoglutarate) dependent dioxygenase family, which requires oxygen and 2-OG for catalytic activity, they are also highly sensitive to alterations in levels of 2-OG or other tricarboxylic acid (TCA) cycle intermediates (Hewitson et al., 2007). HIF levels are therefore likely to be regulated by a complex interplay between oxygen and metabolic signals, which are not fully understood.

Here, we use a forward genetic screen in near-haploid human cells to take an unbiased approach to identify genes that regulate HIF1α (the most widely expressed form of HIF) under aerobic conditions. This type of genetic approach had been restricted to yeast, due to the difficulty in generating bi-allelic mutations in human cells, but was recently circumvented with the generation of the near-haploid human KBM7 cell line (karyotype 25, XY, +8, Ph+), which has been used successfully to identify host restriction factors for pathogens (Carette et al., 2009) and epigenetic regulators (Tchasovnikarova et al., 2015). Using a gene-trap mutagenesis screen in KBM7 cells expressing a sensitive fluorescent HIF1α reporter, we not only identify genes involved in the canonical regulation of HIFs (VHL and PHD2), but also identify two conserved mitochondrial genes, oxoglutarate dehydrogenase (OGDH) and lipoic acid synthase (LIAS), which stabilize HIF1α by preventing its prolyl hydroxylation. Both enzymes are required for correct functioning of the OGDH complex (OGDHC), and disruption of this complex stabilizes HIF1α by conversion of 2-OG to L-2-hydroxyglutarate (L-2-HG). Our studies place OGDHC activity central to HIF1α regulation in aerobic conditions and provide the first evidence that defects in lipoic acid synthesis affect PHD activity.

## Results

### A KBM7 Forward Genetic Screen Identifies OGDH and LIAS as Regulators of HIFα Stability

**Figure 1:**
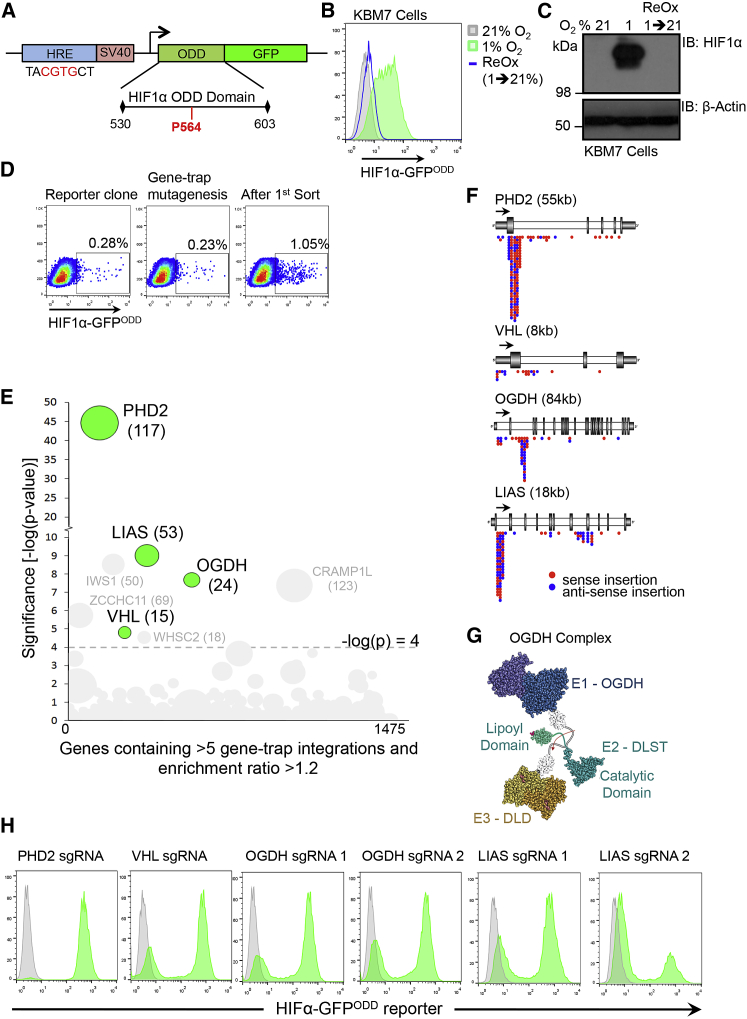
A Human Forward Genetic Screen Identifies OGDH and LIAS as Regulators of HIF1α Stability (A) Schematic of the HIF1α-GFP^ODD^ reporter construct. (B and C) KBM7 cells expressing the HIF1α-GFP^ODD^ reporter were incubated in 21% or 1% oxygen for 24 hr. The cells incubated in 1% oxygen were then exposed to 21% oxygen for 24 hr (reoxygenation). GFP levels were measured by flow cytometry (B) and HIF1α levels by immunoblot (C). (D) KBM7 forward genetic screen with a HIF1α-GFP^ODD^ expressing clone. The cells were mutagenized with the Z-loxP-mCherry gene-trap retrovirus, enriched for GFP^HIGH^ cells by FACS, and insertion sites identified by HiSeq Illumina sequencing. (E) Bubble plot of enriched genes in the GFP^HIGH^ population compared to unsorted mutagenized control KBM7 HIF1α-GFP^ODD^ cells. Bubble size is proportional to the number of independent inactivating gene-trap integrations identified (shown in brackets). Genes that were significantly enriched (>-log(p) 4) and that were successfully validated by CRISPR-Cas9 deletion in HeLa HIF1α-GFP^ODD^ reporter cells are highlighted in green. Four genes (IWS1, ZCCHC11, CRAMP1L, and WHSC2) were enriched for gene-trap insertions but did not pass validation in HeLa HIF1α-GFP^ODD^ reporter cells. (F) Location of the enriched gene-trap insertions in the OGDH, LIAS, VHL, and PHD2 genes (red, sense insertion; blue, anti-sense insertion). The predominance of insertions in the correct orientation at the start of the gene indicates enrichment for gene-trapping mutations. (G) Structure of the OGDH complex (OGDH, DLST, and DLD subunits), showing the region of lipoylation on DLST and its rotation depending on redox cycling by DLD (see also Figure S3B). (H) Validation of the screen using CRISPR-Cas9-targeted depletion (sgRNA) of PHD2, VHL, OGDH, and LIAS in HeLa HIF1α-GFP^ODD^ reporter cells. Two sgRNAs were used for depletion of OGDH and LIAS. GFP reporter levels were measured by flow cytometry. See also Figure S1.

We first developed a sensitive HIF1α reporter for use in the KBM7 forward genetic screen that would reflect the kinetics of endogenous HIF1α stability. A fluorescent construct, consisting of GFP fused with amino acids 530–603 of the HIF1α ODD, was expressed under the control of a minimal HIF-responsive element (HRE) promoter, forming a reporter (HIF1α-GFP^ODD^) that could be induced and degraded in a HIF1α-dependent manner (Figure 1A). The specificity of this reporter was confirmed in KBM7 and HeLa cells lentivirally transduced with the HIF1α-GFP^ODD^ construct. GFP fluorescence was low in cells incubated at 21% oxygen but increased concurrently with HIF1α stabilization when cells were either exposed to hypoxia (1% oxygen) or treated with the PHD inhibitor, dimethyl oxaloylglycine (DMOG) (Figures 1B, 1C, and S1A–S1D). Removing HIF1α-GFP^ODD^ cells from 1% oxygen and re-incubating them at 21% oxygen restored endogenous HIF1α degradation and returned GFP fluorescence to basal levels, thereby confirming that GFP correlated with endogenous HIF1α stability (Figures 1B and 1C). We also ensured that KBM7 cells showed a HIF1α transcriptional response by examining the hypoxic induction of vascular endothelial growth factor (VEGF) and Carbonic Anhydrase 9 (CA9) (Figures S1E and S1F).

We screened for genes that stabilize HIF1α by randomly mutagenizing clonal KBM7 HIF1α-GFP^ODD^ reporter cells with a gene-trapping retrovirus (Figure S1G) and enriching for the rare GFP^HIGH^ cells by a single round of sequential fluorescence-activated cell sorting (FACS) after 8 days (Figure 1D). By mapping gene-trap insertion sites in the GFP^HIGH^ population compared to non-mutagenized controls, we identified several genes that were enriched for inactivating insertions (Figure 1E). We then validated genes identified in the screen using clustered regularly interspaced short palindromic repeats (CRISPR)-Cas9-targeted deletions in HIF1α-GFP^ODD^ reporter HeLa cells. Both PHD2 (the main PHD enzyme responsible for prolyl hydroxylation of HIF1α) and VHL were identified in the screen, validating our genetic approach (Figures 1E and 1F). In addition, we identified two mitochondrial genes, OGDH and LIAS (Figures 1E–1G), which were not only highly enriched for gene-trap insertions but whose CRISPR-Cas9-mediated depletion stabilized the HIF1α-GFP^ODD^ reporter in HeLa cells similarly to depletion of PHD2 and VHL (Figure 1H). OGDH is the E1 component of the OGDHC that converts 2-OG to succinate in the TCA cycle. As this complex requires lipoylation (conjugation with lipoate, the conjugate base of lipoic acid) within the mitochondria by LIAS on the E2 subunit (dihydrolipoyl succinyltransferase [DLST]) for catalytic activity (Figures 1G and S2B), we hypothesized that LIAS and OGDH might regulate HIF1α through a common mechanism.

### Depletion of OGDH and LIAS Leads to the Accumulation of HIF1α in Aerobic Conditions

**Figure 2:**
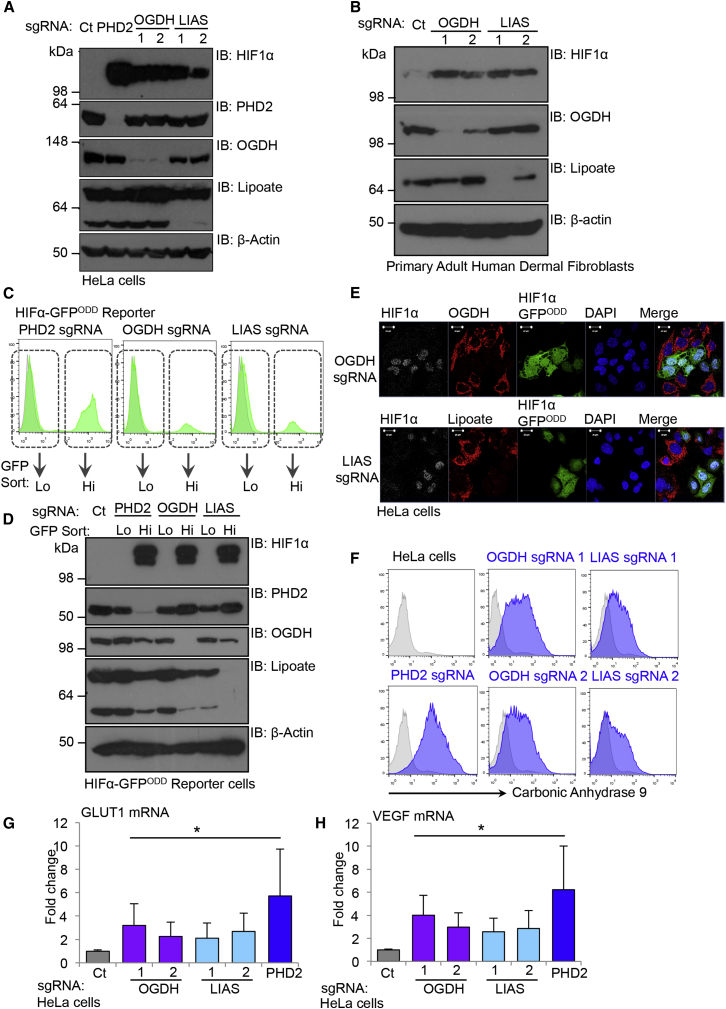
Depletion of OGDH and LIAS Leads to the Accumulation of HIF1α in Aerobic Conditions (A) CRISPR-Cas9 depletion of OGDH and LIAS in HeLa cells. HIF1α levels were measured by immunoblot. sgRNA to PHD2 was used as a control. Lipoate antibody was used to detect lipoylation by LIAS. The lower band relates to DLST lipoylation (see Figure 1G), and higher band relates to PDH complex lipoylation. The different lipoate species are detected with varying efficiency by the lipoate antibody. (B) CRISPR-Cas9 depletion of OGDH and LIAS in primary adult human dermal fibroblasts. Cells were transduced with lentivirus encoding Cas9 and sgRNA to OGDH or LIAS. After 17 days, the cells were lysed and immunoblotted for endogenous HIF1α, PHD2, OGDH, or lipoate. (C and D) HIF1α-GFP^ODD^ HeLa cells were transduced with Cas9 and sgRNA to PHD2, OGDH, or LIAS, sorted by FACS into low (Lo) and high (Hi) GFP populations (C), and immunoblotted for endogenous HIF1α (D). (E) Confocal immunofluorescence microscopy of HIF1α-GFP^ODD^ HeLa cells transduced with lentiviral sgRNA to OGDH and LIAS. (F) Cell surface CA9 was measured by flow cytometry in HeLa cells (gray) depleted of OGDH or LIAS (blue) as described. (G and H) OGDH, LIAS, and PHD2 were depleted in HeLa cells using CRISPR/Cas9-targeted deletions. mRNA from these mixed CRISPR targeted populations was extracted 14 days after lentiviral transduction, and Sybr Green (QIAGEN) qPCR was used to measure the mRNA levels of HIF1α or its target genes, GLUT1 (G) and VEGF (H) (n = 4). Values are mean ± SEM. ^∗^p < 0.05. See also Figures S1 and S2.

CRISPR-Cas9-targeted deletions of OGDH and LIAS in HeLa cells confirmed that depletion of these genes not only stabilized the GFP reporter but also increased endogenous HIF1α levels. HIF1α-GFP^ODD^ reporter or wild-type HeLa cells were transduced with lentiviral constructs encoding Cas9 and single guide RNAs (sgRNAs) targeting OGDH or LIAS and HIF1α levels quantified by immunoblot. Depletion of either LIAS or OGDH increased endogenous HIF1α levels in both wild-type and reporter cells (Figures 2A, S1H, and S1I). The increase in HIF1α was similar to levels observed in cells depleted of PHD2 (Figures 2A and S1H). We also found that depletion of OGDH or LIAS not only stabilized HIF1α in KBM7 and HeLa cells, but also in primary human skin fibroblasts (Figure 2B), suggesting that disruption of these genes increased HIF1α levels through a common cellular mechanism.

FACS of the GFP^LOW^ and GFP^HIGH^ populations confirmed that the increased GFP signal in the reporter cells was due to disruption of LIAS or OGDH, as only the cells deficient of these genes were GFP^HIGH^ in these mixed CRISPR-Cas9-targeted populations (Figures 2C and 2D). Furthermore, by overexpression of OGDH and LIAS designed to be resistant to the relevant sgRNA, we were able to prevent stabilization of HIF1α-GFP^ODD^ and HIF1α upon CRISPR-Cas9 deletion of the endogenous alleles (Figures S1J–S1O).

To determine whether depletion of OGDH or LIAS was associated with a HIF1 transcriptional response, we examined the subcellular localization of HIF1α and measured the expression of HIF1 target genes. Confocal immunofluorescence microscopy confirmed that HIF1α accumulated in the nucleus of HeLa cells depleted for OGDH or LIAS (Figure 2E). This nuclear stabilization was associated with increased cell surface expression of the target gene CA9 (Figure 2F) and the mRNA expression of the HIF1 target genes, GLUT1 and VEGF (Figures 2G and 2H).

The genetic screen suggested that OGDH and LIAS might regulate HIF1α levels through disruption of the OGDHC. Although the E2 (DLST) and E3 (dihydrolipoamide [DLD]) subunits of the OGDHC were not identified in our screen (Figures S2A and S2B), it is unlikely that these screens reach saturation. We therefore asked whether depletion of these genes also increased HIF1α levels and designed sgRNA to target the E2 and E3 subunits in HIF1α-GFP^ODD^ reporter and wild-type HeLa cells. Depletion of either DLST or DLD induced the GFP reporter and increased endogenous HIF1α levels (Figures S2C and S2D), similarly to OGDH and LIAS depletion. Therefore, depletion of any OGDHC component stabilizes HIF1α in aerobic conditions.

### Depletion of OGDH or LIAS Prevents Prolyl Hydroxylation of HIF1α

**Figure 3:**
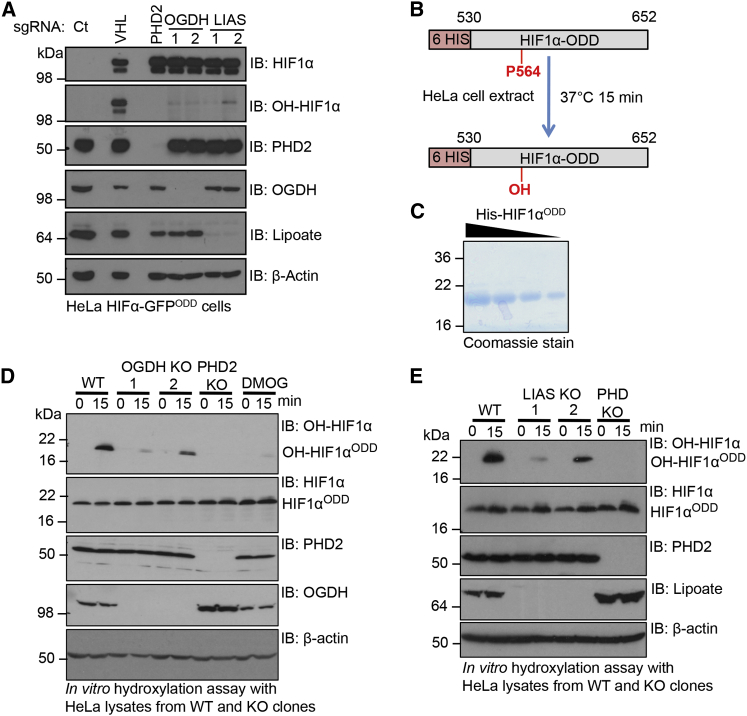
Depletion of OGDH and LIAS Prevents Prolyl Hydroxylation of HIF1α (A) Immunoblots of total HIF1α and prolyl hydroxy-HIF1α (OH-HIF1α) in HeLa HIF1α-GFP^ODD^ cells transduced with lentiviral sgRNA to VHL, PHD2, OGDH, and LIAS. (B) Schematic of in vitro prolyl hydroxylation reaction of HIF1α^ODD^. (C) Coomassie-stained gel of the recombinantly expressed His-HIF1α^ODD^ protein. (D and E) In vitro prolyl hydroxylation of the HIF1α^ODD^ protein using lysates from PHD2, OGDH (D), and LIAS (E) KO clones. See also Figure S3.

As the predominant mechanism for regulating HIF1α levels is through prolyl hydroxylation, we examined whether this was disrupted when OGDH or LIAS were depleted. Using HIF1α-specific antibodies that distinguish between total and hydroxyprolyl levels, we observed that both OGDH and LIAS depletion resulted in the accumulation of HIF1α in a non-hydroxylated form (Figure 3A). Indeed, accumulation of non-hydroxylated HIF1α following disruption of the OGDHC was similar to that seen in cells depleted of PHD2 (Figure 3A). This is in marked contrast to CRISPR-Cas9 depletion of VHL, which acts downstream of prolyl hydroxylation, and therefore causes the accumulation of hydroxylated HIF1α (Figure 3A). Furthermore, we observed no decrease in PHD2 protein levels (Figure 3A), suggesting that disruption of the OGDHC stabilized HIF1α by inhibiting PHD2 activity rather than altering cellular levels of the enzyme.

We also isolated several knockout (KO) clones from the lentiviral CRISPR-Cas9-targeted populations. While prolonged culture of OGDH and LIAS CRISPR-Cas9-targeted cells did result in increased cell death, we were able to isolate several OGDH and LIAS null clones, which proliferated and could be passaged similarly to PHD2 KO cells (Figure S3A). These OGDH and LIAS clones still showed accumulation of HIF1α compared to controls after several passages, albeit to a lesser extent than the PHD2 null clones (Figures S3A–S3C). It was also noteworthy that the OGDH null clones had higher levels of HIF1α compared to the LIAS null clones (Figures S3A–S3C). However, consistent with results from the mixed CRISPR-targeted cells, HIF1α was accumulated in a non-hydroxylated form (Figure S3C).

To directly measure prolyl hydroxylase activity following disruption of the OGDHC, we established an in vitro assay (Figure 3B). Lysates from wild-type HeLa or mixed CRISPR-Cas9-targeted populations for OGDH, LIAS, and PHD2 were incubated with a purified His-tagged HIF1α^ODD^ protein (Figure 3C) and hydroxylation measured using the hydroxyprolyl-specific antibody at 0 and 15 min. The HIF1α^ODD^ protein was rapidly hydroxylated in the wild-type lysate, but reduced hydroxylation of HIF1α^ODD^ was observed in the OGDH-, LIAS-, and PHD2-depleted cell extracts (Figure S3D). Furthermore, in vitro hydroxylation of the HIF1α^ODD^ protein was markedly reduced in all OGDH and LIAS KO lysates compared to the control (Figures 3D and 3E). Thus, disruption of the OGDHC or inhibition of mitochondrial lipoylation stabilized HIF1α by inhibiting PHD enzymatic activity.

### Disruption of the OGDHC Drives the Formation of 2-HG

**Figure 4:**
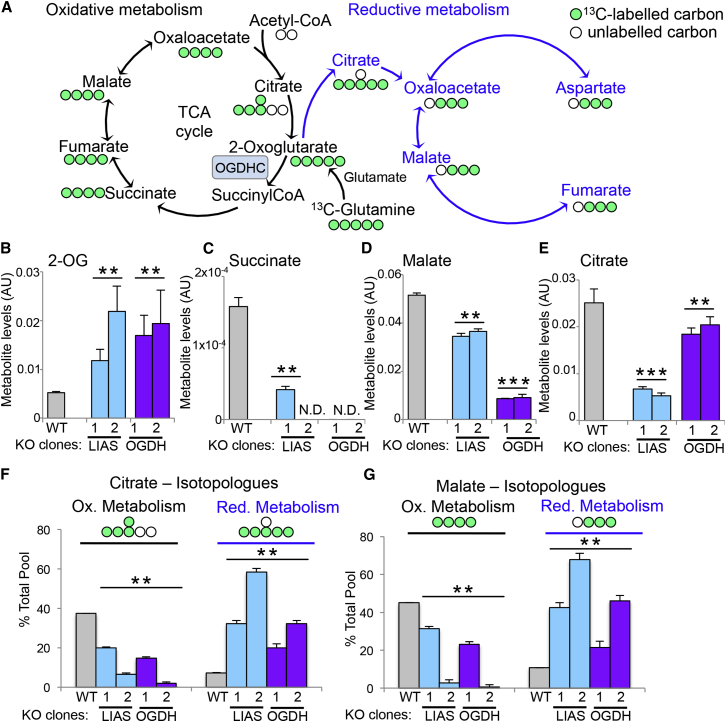
Changes in Cellular Metabolism following Depletion of OGDH or LIAS (A) Schematic of the TCA cycle (black arrows) and reductive metabolism (blue arrows) indicating the number of labeled carbons (green) derived from ^13^C-glutamine. The position of the OGDHC is highlighted. (B–E) Relative intracellular abundance of key metabolites involved in the TCA cycle. (F and G) Isotopologue abundance of m+4 and m+5 citrate (F), and m+4 and m+3 malate (G), measured by ^13^C-glutamine isotope labeling and expressed as a percentage of the total metabolite level. The number of labeled carbons (green) is indicated. n = 5. ^∗∗^p < 0.01 WT compared to KO clones, ^∗∗∗^p < 0.01 OGDH compared to LIAS null clones. ND, not detected. See also Figures S4 and S5.

Small molecule metabolites can inhibit PHD activity in aerobic conditions (Hewitson et al., 2007, Selak et al., 2005). To identify the metabolic changes responsible for decreasing prolyl hydroxylation of HIF1α, we measured metabolite levels in wild-type, PHD2, OGDH, and LIAS null HeLa cells using liquid chromatography-mass spectrometry (LC-MS) and ^13^C-glutamine isotope labeling (Figure 4A). Levels of cellular metabolites in the PHD2 null clones were assessed to account for the metabolic changes caused by HIF1 activation.

Principle component analysis demonstrated that OGDH and LIAS depletion was associated with marked alterations in intracellular metabolites, with low variation between each gene KO (Figures S4A and S4B). This was in contrast to the PHD2 null cells, which did not impact significantly on most cellular metabolites compared to the wild-type cells (Figures S4A and S4B), indicating that activation of HIF1 was not responsible for the metabolic changes seen following OGDH or LIAS depletion. Hierarchical clustering highlighted the different metabolic profiles of the OGDH and LIAS KO cells (Figure S4C), as depletion of OGDH or LIAS impacted on distinct aspects of cellular metabolism, particularly pyruvate metabolism. This was to be expected, as lipoate is also a cofactor for the pyruvate dehydrogenase (PDH) complex; thus, LIAS KO clones have elevated levels of pyruvate and lactate compared to the OGDH clones (Figures S5D–S5F).

The most striking alteration in both the OGDH and LIAS KO clones compared to wild-type cells was near-complete disruption of the TCA cycle at the point of the OGDHC, with accumulation of 2-OG, barely detectable levels of succinate, and decreased levels of malate (Figures 4B–4E). In support to this observation, we observed that ^13^C-glutamine oxidation within the TCA cycle was reduced (Figures 4F and 4G). The ^13^C-glutamine isotope labeling also demonstrated that deletion of OGDH or LIAS promoted reductive metabolism, evidenced by an increase in m+5 citrate, derived from m+5 2-OG (Figure 4F), and m+3 malate, derived from m+5 citrate (Figure 4G). Reduced mitochondrial function was also confirmed by the marked reduction in oxygen consumption rates seen in cells depleted of OGDH or LIAS (Figures S5A–S5F).

The metabolic profile of the OGDH and LIAS null cells suggested that PHD activity must be inhibited despite high levels of 2-OG and low succinate. This was particularly intriguing, as prior studies showed that mutations in other TCA cycle enzymes inhibit PHD activity through the accumulation of TCA intermediates such as succinate (Selak et al., 2005). We therefore sought other mechanisms for regulating PHD activity. We excluded a major role of mitochondrial reactive oxygen species (ROS), as we only observed a small increase in ROS following OGDH or LIAS depletion using the MitoSOX Red mitochondrial superoxide indicator, which was unlikely to be sufficient to stabilize HIF1α (Figures S5G and S5H), and instead focused on shared metabolic consequences of deleting these genes.

**Figure 5:**
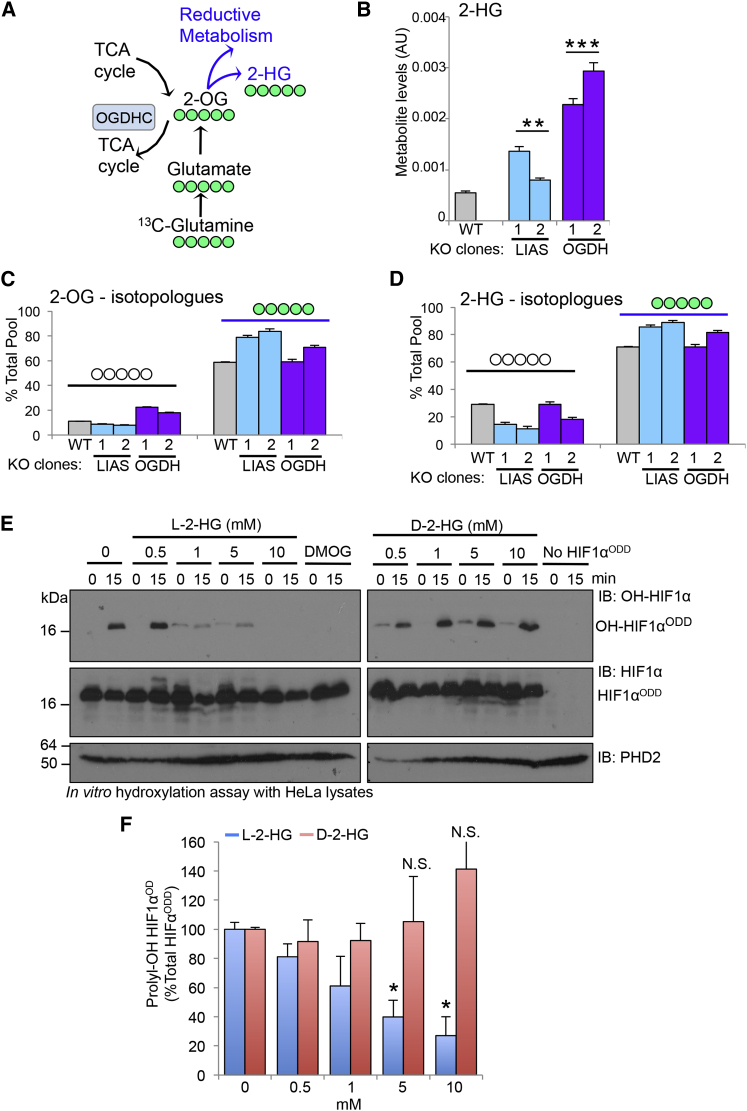
Disruption of the OGDHC Promotes the Formation of 2-HG (A) Schematic of the formation of 2-HG from 2-OG, indicating the number of labeled carbons expected (green) when m+5 2-OG is directly converted to m+5 2-HG following ^13^C-glutamine labeling. (B) Intracellular abundance of 2-HG in the wild-type, KO OGDH, and KO LIAS HeLa clones. (C and D) Levels of m+0 and m+5 2-OG (C) and 2-HG (D) isotopologues measured by ^13^C-glutamine isotope labeling. Five independent cell cultures were measured for each KO clone, and two KO clones were used for each gene. ^∗∗^p < 0.01 WT compared to KO clones, ^∗∗∗^p < 0.01 OGDH compared to LIAS null clones. (E and F) In vitro assay measuring the effect of L- or D-2-HG on prolyl hydroxylation of the HIF1α ODD. Recombinant pure HIF1α^ODD^ protein was incubated with HeLa cell extracts for 0 or 15 min at 37°C, with or without the addition of increasing concentrations of L-2-HG (left) or D-2-HG (right) (0.5–10 mM). Hydroxylation of HIF1α^ODD^ was measured using the hydroxyprolyl-specific antibody (E) and compared to total levels of the HIF1α^ODD^ protein (F), n = 3. Values are mean ± SEM. ^∗^p < 0.05 (L-2-HG treatment compared to control). See also Figure S6.

One metabolite that significantly accumulated in both OGDH and LIAS KO clones compared to the controls was 2-hydroxyglutarate (2-HG) (Figures 5A and 5B), a chiral compound derived from 2-OG (Figures 5C and 5D) that can inhibit 2-OG dioxygenases (Chowdhury et al., 2011, Koivunen et al., 2012, Tarhonskaya et al., 2014b). In support of this finding, we observed decreased activity of the TET 2-OG-dependent dioxygenases (which are inhibited by cellular accumulation of 2-HG [Xu et al., 2011]) by measuring the levels 5-hydroxymethylcytosine (5hmC) in the OGDH- or LIAS-deficient cells (Figures S6A–S6D).

While both L and D enantiomers of 2-HG can be formed in cells, there are conflicting reports as to whether these compounds inhibit or activate PHDs. In vitro studies suggest that L-2-HG is a potential inhibitor of PHD enzymatic activity (Chowdhury et al., 2011), although this has not been shown in cells. D-2-HG, which is mainly formed by mutations in isocitrate dehydrogenase (IDH) (Dang et al., 2009), has been reported to both inhibit and activate prolyl hydroxylase enzymes (Koivunen et al., 2012). We used chiral derivatization to distinguish between the L and D-2-HG enantiomers prior to analysis by LC-MS/MS and observed that while both L-2-HG and D-2-HG levels were increased in OGDH null clones compared to WT and PHD2 KO cells, there was a 10-fold increase in L-2-HG levels, with only a 4-fold change in D-2-HG levels (see Figure S6G). Of note, quantification of 2-HG indicated that this metabolite reaches 0.67 mM (±0.24) in OGDH-deficient cells (Table S1), which is in the region of the IC_50_ for inhibition of PHD by L-2-HG (Chowdhury et al., 2011).

To test whether the 2-HG enantiomers were able to inhibit PHD activity in our experimental model, we measured prolyl hydroxylation of HIF1α^ODD^ in HeLa cell extracts treated with increasing concentrations of L- or D-2-HG (Figures 5E and 5F). Hydroxylation of HIF1α^ODD^ was inhibited with increasing concentrations of L-2-HG, but the addition of D-2-HG had no effect on the hydroxylation of the HIF1α^ODD^ protein (Figures 5E and 5F). Thus, L-2-HG was the predominant enantiomer formed when the OGDHC was disrupted and only L-2-HG had the ability to prevent prolyl hydroxylation of HIF1α.

### Decreasing L-2-HG Levels Restores HIF1α Turnover when 2-OG Accumulates or OGDHC Activity Is Impaired

**Figure 6:**
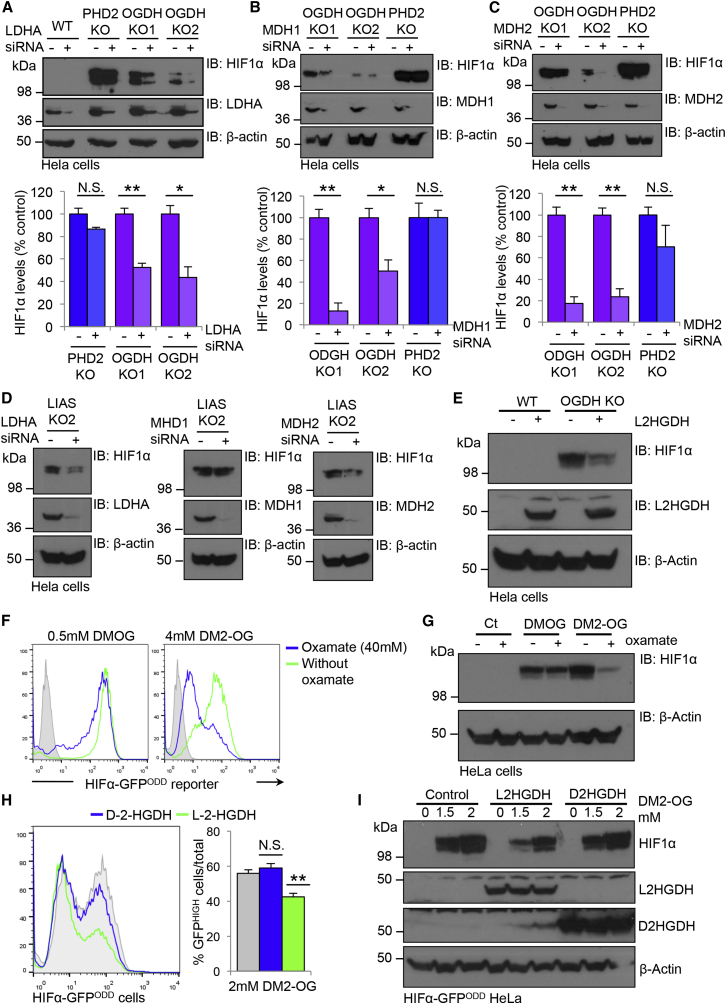
Decreasing L-2-HG Levels Restores HIF1α Turnover when 2-OG Accumulates or OGDHC Activity Is Impaired (A–C) siRNA-mediated depletion of LDHA (A), MDH1 (B), or MDH2 (C) in wild-type HeLa, OGDH null, and PHD2 null clones. siRNA were transfected into OGDH or PHD KO cells and HIF1α levels measured by immunoblot after 72 hr (A–C, top). Densitometry analysis of HIF1α levels (A–C, bottom) (n = 3). (D) siRNA-mediated depletion of LDHA, MDH1, or MDH2 in LIAS null clones. (E) HIF1α levels in OGDH KO clones transduced with or without L-2-HGDH. HIF1α and L-2-HGDH levels visualized by immunoblot. (F and G) HIF1α-GFP^ODD^ reporter (F) or wild-type (G) HeLa cells were treated with 4 mM DM2-OG or 0.5 mM DMOG with or without the addition of 40 mM oxamate for 24 hr. HIF1α levels were measured by GFP levels (F) or by immunoblot (G). (H and I) HIF1α-GFP^ODD^ reporter HeLa cells overexpressing L-2-HDGH or D-2-HGDH were treated with DM2-OG for 24 hr. HIF1α levels were measured by GFP levels (H) or by immunoblot (I). Quantification of mean change in GFP levels is shown (I, right) (n = 4). Values are mean ± SEM. ^∗^p < 0.05. ^∗∗^p < 0.01. ^∗∗∗^p < 0.001. See also Figure S6.

Because L-2-HG is formed from 2-OG by spurious activity of lactate dehydrogenase A (LDHA) and the malate dehyodrogenases (MDH1 and MDH2) (Intlekofer et al., 2015, Oldham et al., 2015), depletion of these enzymes should restore PHD function in the OGDH- or LIAS-deficient cells. We therefore depleted LHDA, MDH1, and MDH2 in the OGDH and LIAS KO clones and measured endogenous HIF1α levels (Figures 6A–6D). Depletion of LDHA, MDH1, or MDH2 all restored HIF1α turnover (Figures 6A–6D). Treating OGDH- or LIAS-deficient cells with the LDHA inhibitor oxamate also restored HIF1α turnover (Figures S6E and S6F) and prevented the accumulation of L-2-HG in OGDH null cells (Figure S6G). Furthermore, we also found that promoting the conversion of L-2-HG to 2-OG by overexpressing the specific L-2-HG dehydrogenase (L-2-HGDH) decreased HIF1α levels in the OGDH KO cells (Figure 6E). Thus, L-2-HG is responsible for the reversible inhibition prolyl hydroxylation of HIF1α.

To determine whether high intracellular levels of 2-OG were sufficient to promote PHD inhibition in aerobic conditions, we examined whether cell-permeable 2-OG stabilized HIF1α. Wild-type and HIF1α-GFP^ODD^ reporter HeLa cells were treated with a cell-permeable 2-OG analog, dimethyl 2-OG (DM2-OG), for 24 hr. HIF1α levels were measured by stabilization of the reporter or by immunoblot, and 2-HG levels by mass spectrometry. DM2-OG treatment increased 2-HG levels in cells from approximately 0.15 mM to 1 mM (Table S1) and stabilized endogenous HIF1α (Figures S6H and S6I) similarly to levels observed with disruption of OGDHC activity. Moreover, HIF1α levels were reversed to near-basal levels following treatment with the LDHA inhibitor oxamate (Figures 6F and 6G). To confirm that HIF1α stabilization was dependent on L-2-HG formation, we overexpressed the enantiomer-specific 2-HG dehydrogenases (L-2-HGDH and D-2-HGDH) in wild-type and HeLa reporter cells treated the cells with DM2-OG. Cells overexpressing L-2-HGDH had reproducibly less HIF1α stabilization compared to wild-type and controls and those expressing D-2-HG (Figures 6H–6J). Together, these experiments show that the accumulation of 2-OG in aerobic conditions is sufficient to promote the formation of L-2-HG, inhibit PHD activity, and stabilize HIF1α.

### Human Homozygous Germline Mutations in Mitochondrial Lipoylation Stabilize HIF1α

To explore the physiological relevance of 2-OG accumulation in aerobic conditions, we focused on several recently described rare inborn errors of metabolism in lipoic acid synthesis. Children with these mutations present with variant non-ketotic hyperglycinaemia (vNKH), a form of Leigh’s syndrome, characterized by severe neurological defects, lactic acidosis, and raised 2-OG and glycine levels (Baker et al., 2014, Mayr et al., 2011). As lipoate is only formed within the mitochondria by LIAS in eukaryotic cells, supplementation with exogenous lipoic acid is not sufficient to treat vNKH and the affected individuals due at a young age (Baker et al., 2014). Interestingly, although the predominant characteristics of vNKH relate to altered metabolism, several features would be in keeping with HIF1 activation, including angiogenesis in the neurological lesions, cardiomyopathies, and the development of pulmonary hypertension (Ahting et al., 2015).

**Figure 7:**
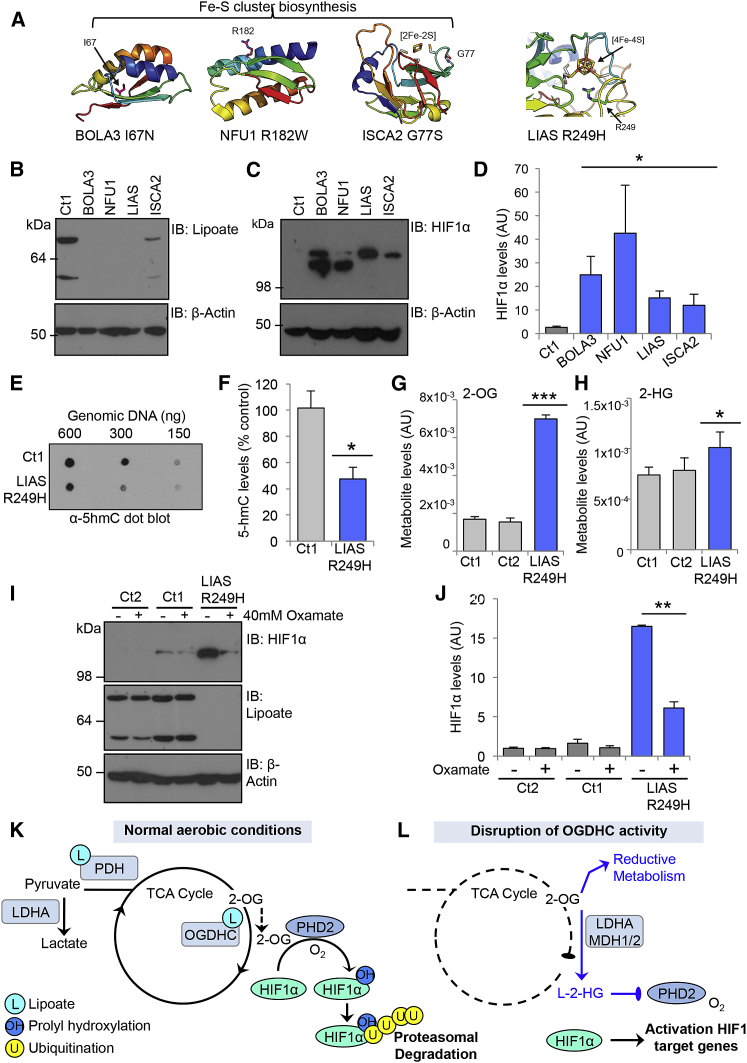
Human Germline Mutations in Mitochondrial Lipoylation Stabilize HIF1α (A) Structural modeling of mutations in the Fe-S cluster biogenesis genes (BOLA3, NFU1, and ISCA2) and LIAS. Germline homozygous mutated residues are indicated, and Fe-S binding sites are highlighted. (B–D) Levels of HIF1α and lipoylation in skin fibroblasts from patients with mutations in proteins required for lipoic acid synthesis. Lipoate and total HIF1α levels were analyzed by immunoblot. HIF1α levels were also quantified from four separate experiments by densitometry analysis (D). Different isoforms of HIF1α are likely to account for the small changes in HIF1α migration observed in patient fibroblast lines (C). (E and F) TET enzyme activity in control or LIAS R249H mutant fibroblasts. Total genomic DNA levels were measured by methylene blue staining, and the relative levels of 5hmC quantified by densitometry (F) (n = 4). (G and H) Relative intracellular abundance of 2-OG (G) and 2-HG (H), in control and LIAS R249H mutant fibroblasts measured by LC-MS as described. (n = 5). (I and J) Oxamate treatment prevents HIF1α accumulation in LIAS mutant fibroblasts. Control or LIAS R249H fibroblasts were treated with 40 mM oxamate for 24 hr and HIF1α levels measured by immunoblot (I). Fibroblasts from two unrelated patients were used as controls (Ct1 and Ct2). Quantification of HIF1α levels from three separate experiments is shown in (J). ^∗^p < 0.05, ^∗∗^p < 0.01, ^∗∗∗^p < 0.001. (K and L) Model for the mechanism of HIF1α stabilization following disruption of the OGDHC by depletion of OGDH or decreased mitochondrial lipoylation. In resting cells, 2-OG is mainly utilized in oxidative metabolism and HIF1α is hydroxylated by PHD2, driving its rapid ubiquitination and proteasomal degradation (K). When OGDHC activity is reduced, the accumulation of 2-OG drives reductive metabolism and the formation of L-2-HG, which inhibits PHD activity, thereby stabilizing HIF1α in aerobic conditions (L). See also Figure S7.

We obtained skin fibroblasts from four vNKH patients with previously described or causative homozygous mutations in different genes involved in lipoic acid synthesis: LIAS, NFU1 (NFU1 Iron-Sulfur Cluster Scaffold), ISCA2 (Iron-Sulfur Cluster Assembly 2), and BOLA3 (BolA Family Member 3) (Figure 7A) (Ahting et al., 2015, Haack et al., 2013, Mayr et al., 2011). LIAS incorporates an iron-sulfur (4Fe-4S) cluster for catalytic activity, and it has recently been shown that mutations in Fe-S biogenesis genes (BOLA3, NFU1, and ISCA2) reduce lipoate formation and result in vNKH (Baker et al., 2014). Homozygous mutations in LIAS, BOLA3, and NFU1 not only showed marked defects in lipoylation compared to control fibroblasts (Figure 7B), but also had reduced OCRs (Figure S7A), similarly to rates observed following LIAS depletion in HeLa cells (Figures S5A–S5F). Mitochondrial lipoylation was still detected in the mutant ISCA2 cells, which had an OCR similar to the control fibroblasts, suggesting that homozygous mutations in ISCA2 result in a milder phenotype. However, all homozygous mutations in lipoic acid synthesis demonstrated higher levels of HIF1α compared to control fibroblasts (Figures 7C and 7D). This increase in HIF1α was associated with an increase in mRNA expression of GLUT1, without significant changes in HIF1α transcript levels (Figures S7B and S7C), consistent with defective lipoylation inhibiting PHD activity under aerobic conditions. Moreover, 5hmC levels were reduced by approximately 50% in the LIAS R249H fibroblasts compared to controls (Figures 7E and 7F), consistent with defects in lipoylation also affecting the TET 2-OG-dependent dioxygenases.

To determine whether L-2-HG derived from 2-OG was responsible for stabilizing HIF1α in the lipoate-deficient cells, we measured intracellular metabolite abundance in the control and mutant LIAS fibroblasts. Levels of 2-OG and 2-HG were increased in the LIAS R249H mutant fibroblasts compared to the controls (Figures 7G and 7H). Furthermore, we observed a general decrease in the levels of TCA intermediates consistent with a reduction in oxidative metabolism (Figures S7D and S7E), which were similar to the metabolite changes seen in the HeLa LIAS null clones (Figure 4). In addition, 2-HG levels appeared to correlate with the degree of HIF1α stabilization observed in the LIAS mutant fibroblasts compared to the HeLa LIAS or OGDH KOs, with 2-HG and HIF1α levels highest in the OGDH KO clones (Figure 5B).

We next examined whether decreasing L-2-HG formation restored HIF1α turnover. Mutant LIAS R249H or control fibroblasts were treated with 40 mM oxamate for 24 hr and HIF1α levels measured by immunoblot. LDHA inhibition reproducibly decreased HIF1α levels in the LIAS R249H fibroblasts compared to the controls (Figures 7I and 7J). Similar findings were observed with oxamate treatment of the BOLA3, NFU1, and ISCA2 mutant fibroblasts (Figure S7F), consistent with LDHA inhibition restoring HIF1α turnover. We also examined whether oxamate was able to restore HIF1α turnover in the LIAS mutant cells at lower oxygen tensions. Control or LIAS R249H fibroblasts were incubated in 6% oxygen and treated with oxamate for 24 hr. While HIF1α levels were still reduced in the oxamate-treated LIAS R249H fibroblasts at 6% oxygen, we now observed that oxamate treatment also decreased the levels of HIF1α in the control cells (Figure S7G), supporting the notion that 2-OG and L-2-HG levels may contribute to the homeostatic regulation of PHD activity. Thus collectively, these data confirm a role for mitochondrial enzyme lipoylation and the OGDHC in regulating prolyl hydroxylation of HIF1α through the formation of L-2-HG (Figures 7K and 7L).

## Discussion

Using an unbiased forward genetic approach, we identify that the OGDHC is central to the regulation of PHD activity in aerobic conditions. This was unexpected, as intracellular accumulation of 2-OG was unlikely to inhibit PHD activity, and prior reports show that cell-permeable 2-OG can overcome succinate-mediated inhibition of HIF1α prolyl hydroxylation (MacKenzie et al., 2007). Instead, we uncover that PHD activity is inhibited when 2-OG accumulates, through the formation of L-2-HG. The biological implications of these findings are diverse, as disrupting the OGDHC itself, or genes involved in Fe-S and lipoate biosynthesis, all stabilize HIF1α through the same mechanism. Although 2-HG is increasingly recognized as a major metabolic regulator in tumors (Kaelin, 2011), the relative importance of this molecule in regulating PHDs was controversial, particularly as it was thought that 2-HG is unlikely to inhibit PHD activity without a concurrent decrease in 2-OG levels (Xu et al., 2011). We now show that L-2-HG is not only able to inhibit PHD2, but is also derived from accumulated 2-OG within cells in aerobic conditions. These findings are consistent with the formation of L-2-HG from 2-OG in hypoxia (Intlekofer et al., 2015, Oldham et al., 2015) and suggest a more general role for 2-OG metabolism in regulating prolyl hydroxylases.

Our metabolic and biochemical studies show that L-2-HG is the predominant enantiomer formed when the OGHC is disrupted. Moreover, prolyl hydroxylation of HIF1α was only inhibited by L-2-HG and not D-2-HG, consistent with prior reports (Chowdhury et al., 2011). Whether D-2-HG can activate HIF1α (Koivunen et al., 2012) is unclear from our studies. Although we observed a small increase in hydroxylation of the HIF1α protein following the addition of D-2-HG, Tarhonskaya et al. show that this increase is due to non-enzymatic oxidation of 2-HG when iron and reducing agents are added to the reaction (Tarhonskaya et al., 2014a). It is noteworthy that exogenous iron and ascorbate were not required for our in vitro assay. Irrespective of these findings, the dominant effect observed in our experiments seems to be PHD inhibition by L-2-HG when OGDHC activity is reduced.

We observed that oxamate decreased HIF1α levels in primary fibroblasts incubated in 6% oxygen, a concentration representative of physiological oxygen levels in tissues. It is therefore possible that L-2-HG formation may serve a homeostatic role in regulating PHD activity in both aerobic and anaerobic conditions. Indeed, further support for a feedback loop is suggested by the ability of HIF1α to promote the proteasome-mediated degradation of a short OGDH isoform (OGDH2) and decrease OGDHC activity (Sun and Denko, 2014). Although we observed lower levels of the major OGDH isoform in PHD2 null cells and decreased mitochondrial lipoylation (Figure 2D), we did not find any significant alterations in OGDHC complex activity or 2-OG levels in the PHD2 KO cells compared to wild-type HeLa cells (Figure S6). It will be of interest in further studies to determine the relative importance of this potential feedback mechanism in physiological contexts.

The finding that HIF1α is elevated in patient cells with homozygous mutations in lipoic acid synthesis highlights the potential role of this transcription factor in these conditions. Indeed, a recent genome-wide screen identifies hypoxia and VHL inhibition as a potential therapy for mitochondrial diseases presenting with Leigh syndrome (Jain et al., 2016). It is therefore possible that HIF1 activation serves as a protective physiological response in patients with defective lipoic acid synthesis. Homozygous mutations in lipoic acid synthesis are rare, and mostly lethal at a young age, therefore certain features associated with HIF1 activation, such as tumor formation, may not be expected. Moreover, although the patients did not have an erythrocytosis, the occurrence of vascular proliferation in the neurological lesions and the development of pulmonary hypertension in some of the patients with lipoate deficiency is intriguing and warrants further investigation.

Heterozygous germline mutations in genes involved in lipoic acid synthesis or the OGDHC have not been characterized, but it is in these patients that loss of heterozygosity may promote tumor formation as has been shown for heterozygous mutations in the TCA cycle enzymes succinate dehydrogenase (SDH) and fumarate hydratase (FH) (Pollard et al., 2007, Selak et al., 2005). Indeed, while the tumorigenic role of 2-OG-dependent dioxygenases is debated, it will be of interest to determine whether OGDHC activity and lipoate formation influence the enzymatic activity of other 2-OG-dependent dioxygenases, similarly to the PHDs and TET enzymes shown here. Indeed, several somatic mutations in LIAS or OGDH have been collated (Catalogue of Somatic Mutations in Cancer [COSMIC] database) (Forbes et al., 2010), and a number of the LIAS mutations are similar to the known homozygous mutations, which tend to occur around the Fe-S cluster coordination sites. Whether these mutations promote HIF1 stabilization, histone modification, and tumor formation will be important to address in future studies.

## Experimental Procedures

### Cell Culture and Reagents

KBM7 cells were maintained in Iscove’s Modified Dulbecco’s Medium (IMDM, GIBCO) supplemented with 10% fetal calf serum (FCS) and 1% penicillin/streptomycin. HeLa and HEK293ET cells were maintained in Dulbecco’s Modified Eagle’s Medium (DMEM, GIBCO) supplemented with 10% FCS. Primary skin fibroblasts were also cultured in DMEM, but supplemented with 20% FCS. Fibroblast cell lines from patients with homozygous mutations in lipoic acid synthesis are detailed in Supplemental Information. Hypoxic cell culture was performed in a Whitley H35 Hypoxystation (Don Whitley Scientific) at 37°C/5% CO_2_ plus either 1% O_2_/94% N_2_ or 6% O_2_/89% N_2_.

A complete list of plasmids, reagents, and antibodies used are detailed in Supplemental Information.

### Lentiviral/Retroviral Production and Transduction

Lentivirus was produced by triple-transfection of HEK293ET cells with the appropriate lentiviral transgene vector and the packaging vectors pCMVR8.91 (gag/pol) and pMD.G (VSVG). Transfection was performed using Trans-IT 293 reagent (Mirus) with cells at 70%–80% confluency in 6-well plates. Viral supernatant was harvested at 48 hr, filtered through a 0.45 μm filter, and frozen at −80°C until required. For transduction, cells were seeded to 24-well plates in 500 μL culture medium. 500 μL viral supernatant was added to each well and plates centrifuged at 1,800 rpm, 37°C for 1 hr. Plates were then incubated for 3 hr before an additional 1 mL fresh medium was added to each well. Cells were expanded and antibiotic selection applied from 48 hr if required. The Z-loxP-mCherry gene-trap retrovirus was produced as for the lentivirus supernatants but with the appropriate packaging vectors (pMD.Gag.Pol and pMD.VSVG).

### Flow Cytometry

2 × 10^5^ cells per sample were washed in 3 mL ice-cold PBS in 5 mL round-bottom polystyrene tubes and resuspended in 200 μL PBS/1% formaldehyde prior to analysis on a FACScalibur (GFP, AF488, PI, AF647) or BD Fortessa (GFP, AF488, mCherry, AF568, AF647). For cell-surface staining, cells were washed in 3 mL ice-cold PBS, resuspended, and incubated at 4°C for 30 min with the primary antibody. Samples were then washed with PBS and incubated with an appropriate secondary antibody at 4°C for 30 min.

### Forward Genetic Screen in Near Haploid KBM7 Cells

The KBM7 forward genetic screen was carried out as described by Tchasovnikarova et al. (2015), with some modifications. 1 × 10^8^ clonal KBM7 cells expressing the HIF1α-GFP^ODD^ reporter were transduced with the retroviral Z-loxP-mCherry gene-trap supernatant plus 10 μg/mL hexadimethrine bromide (Polybrene) at 1,800 rpm (37°C) for 1 hr. Cells were then incubated for 3 hr at 37°C before the addition of 500 μL fresh IMDM. Transduction efficiency was measured by flow cytometry after 72 hr.

Cells were enriched for mutagenized GFP^HIGH^ cells by one round of FACS at day 8. 24 hr prior to sorting the cells were purified by centrifugation for 20 min at 1,800 rpm on a Lympholyte cell separation density gradient (Cedarlane Labs) to remove debris. The cells were then resuspended in PBS supplemented with 2% FCS and 10 mM HEPES for sorting. Selected cells were collected into IMDM with 50% FCS and 10 mM HEPES. Cells were expanded for 4 days prior to lysis. Gene-trap integration sites were identified using a PCR-based protocol as described by Tchasovnikarova et al. (2015).

### CRISPR-Cas9-Targeted Deletions

Gene-specific CRISPR sgRNA sequences were selected from the GeCKO v2 library (Sanjana et al., 2014) and used to generate sense and antisense oligonucleotides, with 5′ CACC and 3′ CAAA overhangs respectively. These sgRNA were ligated into the LentiCRISPRv2 vector according to published methods (Sanjana et al., 2014). An additional guanosine base was included in some cases at the beginning of the sgRNA sequence to improve transcription from the U6 promoter. CRISPR lentivirus production and transduction were performed as described. Transduced cells were selected by puromycin treatment. CRISPR-transduced cells were generally cultured for 9–10 days prior to subsequent experiments to allow sufficient time for depletion of the target protein. KO clones were isolated from the sgRNA-targeted populations by serial dilution cloning and immunoblot. The full list of sgRNAs used is detailed in the Supplemental Information.

### Metabolomic Analyses

HeLa cells were seeded to 6-well plates at 3.5 × 10^5^ cells per well in DMEM 24 hr prior to harvesting. For carbon flux analysis, the cells were cultured in glutamine-free DMEM supplemented with 10% FCS and 4 mM ^13^C_5_ L-glutamine (Cambridge Isotope Laboratories) for 24 hr. Metabolites were extracted by washing the cells in ice-cold PBS before adding extraction buffer (50% methanol, 30% acetonitrile, 20% H_2_O, 100 ng/mL HEPES) and incubating the plates for 15 min over dry ice and methanol. The cells were then transferred to 1.5 mL Eppendorf tubes and incubated on dry ice for 5 min. Tubes were transferred to a thermomixer at 4°C and shaken at 1,400 rpm for 15 min before centrifuging at 14,000 rpm, 4°C for 10 min. Finally, the supernatants were transferred into autosampler vials and stored at −80°C prior to Liquid Chromatography Mass Spectrometry (LC-MS) analysis (detailed in the Supplemental Information).

### Immunoblotting

Cells were lysed in an SDS lysis buffer (1% SDS, 50 mM Tris [pH 7.4], 150 mM NaCl, 10% glycerol, and 5 μL/mL Benzonase nuclease) for 10 min before heating at 70°C for 10 min. Proteins were separated by SDS-PAGE, transferred to PVDF membranes, probed with appropriate primary and secondary antibodies, and developed using SuperSignal West Pico or Dura Chemiluminescent Substrates (Thermo Scientific).

### 5hmC DNA Dot Blotting

Genomic DNA was extracted from cultured cells using the Gentra Puregene kit (QIAGEN). Genomic DNA dot blotting for 5hmC levels was performed as previously described (Szulwach et al., 2011) with some modifications. Briefly, the genomic DNA was denatured in 10 mM EDTA 0.4 M NaOH at 100°C for 10 min. 2-fold dilutions of denatured DNA were spotted onto Hybond NX membranes (GE Healthcare), allowed to dry, and rinsed with 2× SCC buffer. The DNA was UV crosslinked to the membrane, blocked with 1% BSA and 5% milk powder, and probed for 5hmC with a rabbit polyclonal antibody (Active Motif). The total DNA levels were quantified by methylene blue staining, and relative densitometry measured using ImageJ.

### Immunofluorescence

HeLa cells were plated on glass coverslips overnight prior to staining. Media was removed by three PBS washes prior fixation with PBS/4% formaldehyde. Cells were permeabilized with 0.3% Triton X-100 and then blocked with 3% BSA. Primary and secondary antibodies were incubated at desired concentrations, before three final washes and mounting to microscope slides using ProLong Gold antifade with DAPI. Imaging was performed on a Zeiss LSM880 confocal microscope.

### qPCR

Total RNA was extracted using the RNeasy Plus minikit (QIAGEN) following manufacturer’s instructions and then reversed transcribed using Super RT reverse transcriptase (HT Biotechnology). PCR was performed on the ABI 7900HT Real-Time PCR system (Applied Biosystems) using SYBR Green Master mix (Applied Biosystems). Reactions were performed with 125 ng of template cDNA. Transcript levels of genes were normalized to a reference index of housekeeping genes (GAPDH and RPS2).

### In Vitro Hydroxylation Assay

To form the HIF1α^ODD^ protein, we expressed a His-tagged protein corresponding to residues 530–652 of human HIF1α in BL21 *E. Coli* and purified using a NiNTA column on an Äkta Pure FPLC (GE Healthcare). Protein purity was assessed by SDS-PAGE and Coomassie staining, and the HIF1α^ODD^ protein was dialyzed into 20 mM Tris (pH 7.4), 150 mM NaCl with 1 mM DTT. The HeLa cell extract was prepared from 1 × 10^8^ cells lysed in 2 mL reaction buffer (RB: 20 mM HEPES [pH 7.5], 5mM KCl, 1.5 mM MgCl_2_) followed by two cycles of freeze/thaw in an ethanol/dry ice bath. The lysates were passed eight times through a 21G needle, followed by two passes through a 26G needle before centrifugation (17,000 × *g* 4°C, 30 min). The supernatants were aliquoted and stored at −80°C. The hydroxylation assay was performed by incubating 10 μM HIF1α^ODD^ with 50 μL HeLa cell extract in RB for 15 min at 37°C. The reaction was stopped by addition of SDS loading buffer, and the proteins separated by SDS-PAGE. Hydroxylation was measured using the HIF prolyl hydroxylation specific antibody. Measurements of DMOG and 2-HG inhibition of HIF1α hydroxylation were performed similarly, except the lysate was pre-incubated with the compounds for 10 min at 37°C before the addition of the HIF1α^ODD^ protein.

### siRNA-Mediated Depletion

siRNA SMARTpools for LDHA, MDH1 or MDH2 (Dharmacon) were transfected into HeLa cells using Oligofectamine Transfection Reagent (Invitrogen) according to the manufacturer’s protocol. Cells were harvested after 72 hr for further analysis by immunoblot.

### Statistical Analyses

Data were expressed as mean ± SEM and p values were calculated using two-tailed Student’s t test for pairwise comparisons, unless otherwise stated. Except for metabolomic experiments, no randomization or blinding was performed. No statistical method or power analysis was used to predetermine sample size.

## Author Contributions

S.P.B., G.L.G., C.F., and J.A.N. designed the studies. S.P.B., G.L.G., A.S.H.C., I.T.L., and J.A.N. performed the experiments. S.P.B. and J.A.N. wrote the manuscript. A.S.H.C. and C.F. designed and performed the metabolomic studies. R.T.T. and P.J.L. contributed to the experimental design of the screen and supported the data analysis. G.D. supported the data analysis. P.F. characterized the ISCA2 mutation. R.B.D. undertook the structural modeling of the proteins.

## Supplemental Information

Document S1. Supplemental Experimental Procedures, Figures S1–S7, and Table S1

Document S2. Article plus Supplemental Information
