# Supplementary material for: Mitochondrial Protein Lipoylation and the 2-Oxoglutarate Dehydrogenase Complex Controls HIF1α Stability in Aerobic Conditions
Source: Cell Metab. 2016 Nov 8;24(5):740–52. doi: 10.1016/j.cmet.2016.09.015 (PMC5106373; doi:10.1016/j.cmet.2016.09.015)
Supplement: Document S1. Supplemental Experimental Procedures, Figures S1–S7, and Table S1 [file mmc1.pdf]

Cell Metabolism, Volume *25*

Supplemental Information

Mitochondrial Protein Lipoylation

and the 2-Oxoglutarate Dehydrogenase Complex

Controls HIF1α Stability in Aerobic Conditions

Stephen P. Burr, Ana S.H. Costa, Guinevere L. Grice, Richard T. Timms, Ian T. Lobb, Peter Freisinger, Roger B. Dodd, Gordon Dougan, Paul J. Lehner, Christian Frezza, and James A. Nathan

SUPPLEMENTARY INFORMATION:

Figure S1: Validation of the HIF1α-GFP^{ODD} reporter, mutagenesis of the KBM7 cells, and reconstitution of the OGDH or LIAS deficient cells (related to Figures 1 and 2).

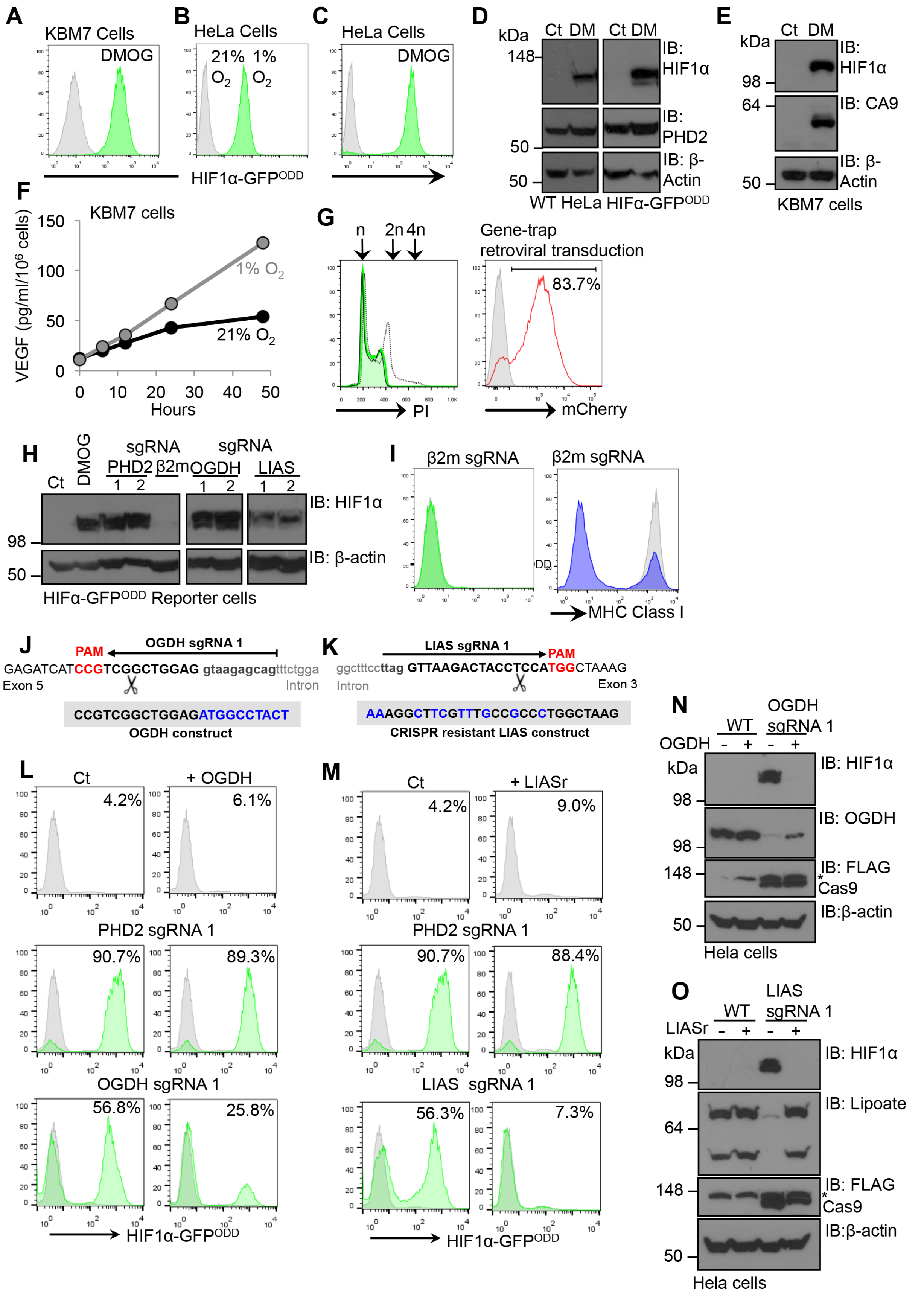

A-C, HIF1α-GFP^{ODD} reporter levels in KBM7 (**A**) and HeLa (**B, C**) cells exposed to 1% oxygen (**B**) or treated with 0.5 mM DMOG for 18 h (**A, C**). **D,** Immunoblot for HIF1α and PHD2 levels in wildtype or HIF1α-GFP^{ODD} reporter HeLa cells treated with DMOG (DM) compared to untreated controls (Ct). **E, F,** Analysis of HIF1 transcriptional response in KMB7 cells measured by immunoblot for carbonic anhydrase 9 (CA9) levels (**E**), or by VEGF ELISA in supernatants following incubation in 21% or 1% oxygen for the indicated times (**F**). **G,** HIF1α-GFP^{ODD} KBM7 cells were confirmed to be near-haploid using Propidium Iodide (PI) staining (left, solid black line = haploid WT KBM7 cells, green = HIF1α-GFP^{ODD} KBM7 cells, dotted black = mixed haploid/diploid WT KBM7 cells), and mutagenised with the Z-loxP-mCherry gene-trap retrovirus (right). Approximately 80% of the cells were transduced with the virus. **H, I,** HIF1α-GFP^{ODD} reporter cells were lentivirally transduced with Cas9 and sgRNA to PHD2, OGDH, LIAS or β2 microglobulin (β2m). sgRNA-mediated depletion of β2 microglobulin (β2m) was used as a negative control. HIF1α levels were measured by immunoblot (**A**). Flow cytometry analysis of cell surface MHC Class I levels confirmed β2m depletion (**B,** right). CRISPR-Cas9 depletion of β2m had no effect on GFP levels in the HIF1α-GFP^{ODD} reporter cells (**B**, left). **J, K,** Schematic of the OGDH and LIAS sgRNA designed to generate indels in endogenous OGDH (**J**) and LIAS (**K**). The protospacer adjacent motif (PAM) and Cas9 cleavage sites are highlighted. Overexpressed OGDH or LIAS (grey) are not efficiently cut by Cas9 as the OGDH guide binds to an intronic region, and silent mutations were incorporated into the LIAS construct (LIASr) to prevent sgRNA binding (bases not present in sgRNA are highlighted in blue). **L,M** Control (Ct) HIF1α-GFP^{ODD} reporter cells, or those stably expressing exogenous OGDH (**L**, right panels) or LIASr (**M**, right panels) were transduced with sgRNA to PHD2, OGDH or LIAS (shaded green). GFP accumulation was measured by flow cytometry after 10 days (% GFP^{HIGH} are shown). **N, O** Endogenous HIF1α levels in wildtype HeLas, or those overexpressing exogenous OGDH or LIASr, after CRISPR-Cas9 depletion of OGDH (**L**) or LIAS (**M**). FLAG-Cas9 levels measured by immunoblot. **non-specific band.*

Figure S2: CRISPR/Cas9-mediated depletion of DLST and DLD stabilises HIF1α (related to Figure 2).

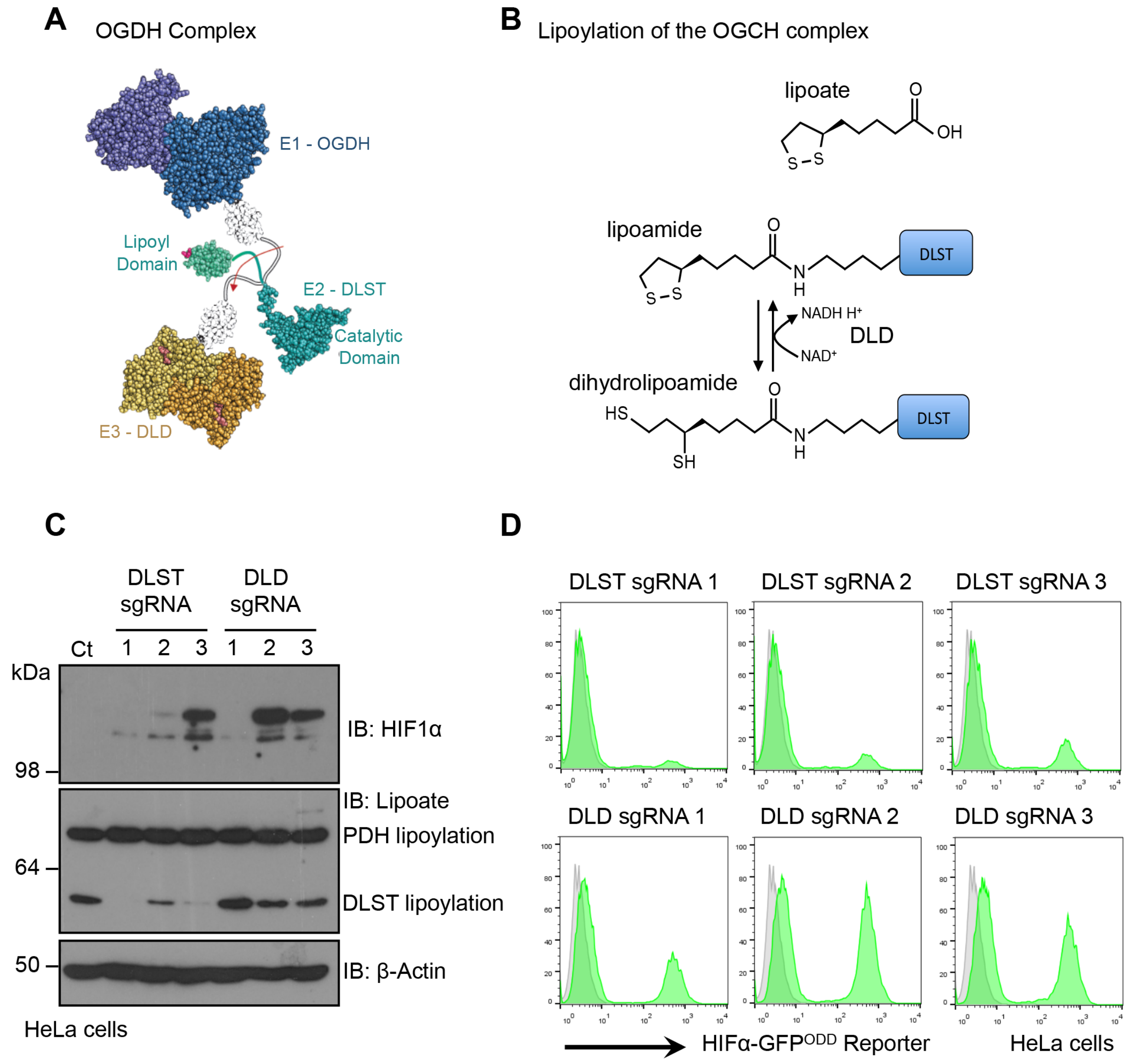

A, B, Lipoylation of the OGDHC. Structure of the OGDHC, indicating the position of the lipoyl domain on DLST (**A**). Lipoylation involves the conjugation of the lipoate molecule to a lysine residue within DLST, forming the lipoamide moiety (**B**). Catalytic activity of the OGDHC requires the formation of dihydrolipoamide on DLST, which is converted back to lipoamide by DLD in a NADH redox reaction (**B**). **C** and **D,** Wildtype (**C**) or HIF1α-GFP^{ODD} reporter HeLas (**D**) were transduced with sgRNA/Cas9 to DLST or DLD. 10 days post transduction, endogenous HIF1α and HIF1α-GFP^{ODD} reporter fluorescence were measured by immunoblot or flow cytometry respectively.

Figure S3: OGDH and LIAS depletion decreases PHD hydroxylation (related to Figure 3).

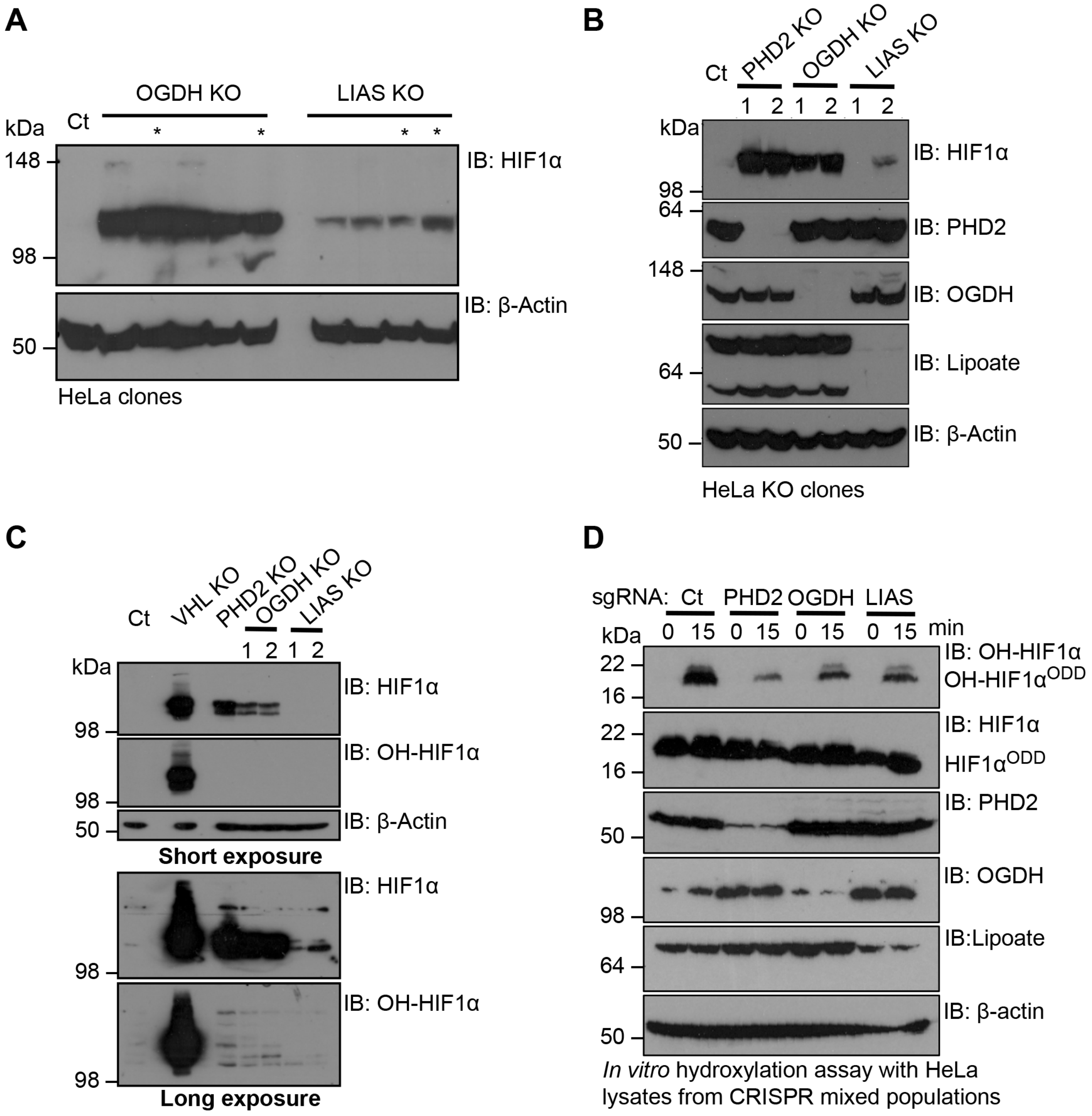

A-C, PHD2, OGDH and LIAS null cells were isolated from HeLa cells transduced with Cas9 and specific sgRNAs by serial dilution cloning. Immunoblot showing HIF1α levels in several OGDH and LIAS sgRNA target clones (**A**). Confirmation that two representative KO clones for each gene (* in **A**) are deficient for OGDH and LIAS (**B**). Hydroxylated HIF1α levels in the PHD2, OGDH or LIAS null clones measured by hydroxy-HIF1α specific antibody (**C**). **D**, *In vitro* hydroxylation of the HIF1α^{ODD} protein using HeLa cell extracts from PHD2, OGDH and LIAS CRISPR-Cas9 targeted populations.

Figure S4: Metabolic analysis of PHD2, OGDH and LIAS deletion in HeLa cells and their effect on pyruvate metabolism (related to Figure 4).

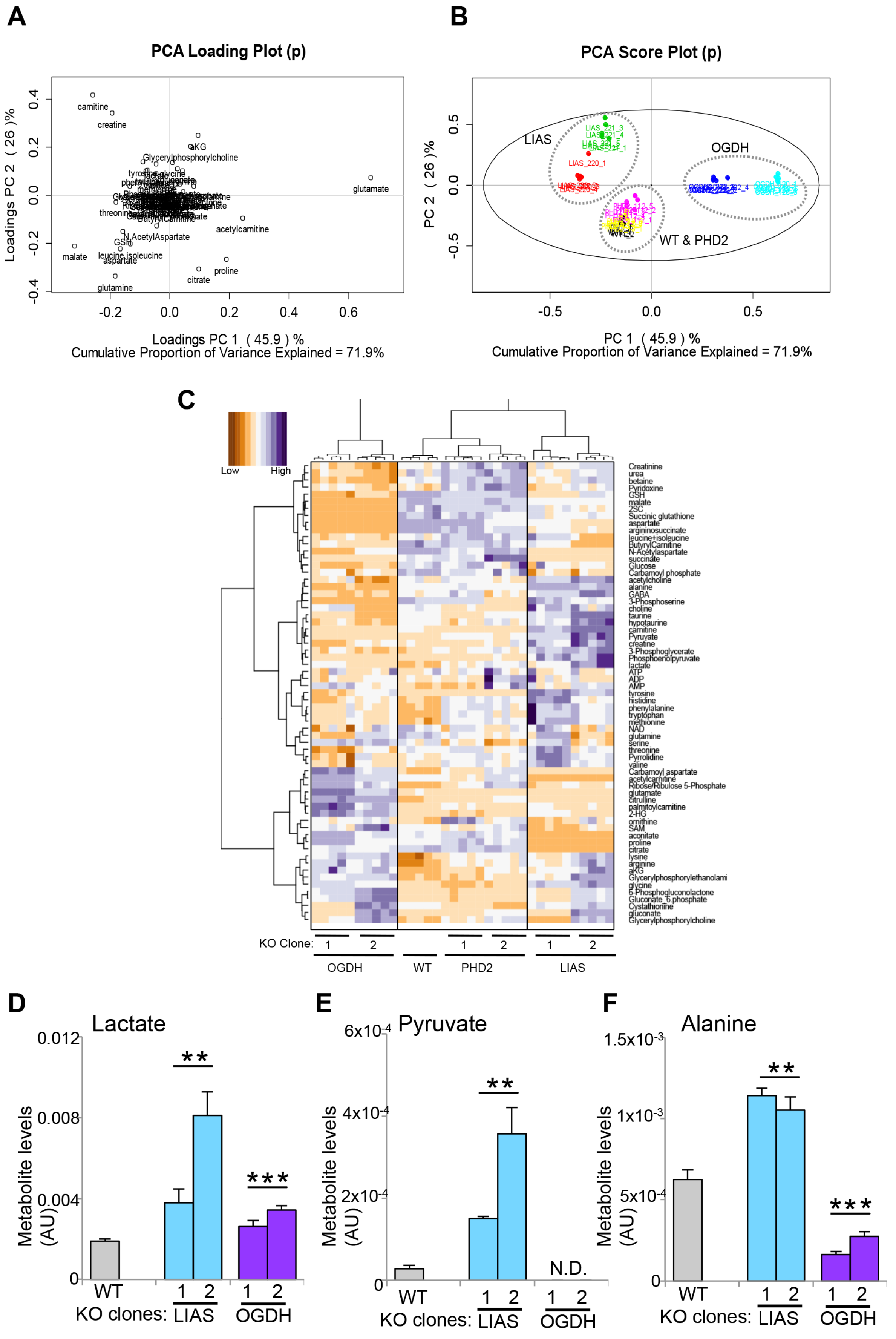

A, Principal Components Analysis loading plot of intracellular polar metabolites contributing to separate HeLa cell extracts from PHD2, OGDH and LIAS CRISPR-Cas9 targeted populations. **B**, Score plot of each five replicates per clone and condition (colour coded) from one experiment. 'Variance Explained' refers to the percentage of total variation observed between samples that can be attributed to separation along a given principal component. **C-E,** Total cellular levels of lactate (**C**), pyruvate (**D**) and alanine (**E**) in wildtype, OGDH KO and LIAS KO HeLa cells measured by LC-MS. Five independent cell cultures were measured for each KO clone, and two KO clones were used for each gene. **P<0.01 WT compared to KO clones, ***P<0.01 OGDH compared to LIAS null clones. N.D= not detected. **C**, Unsupervised hierarchical clustering of metabolite levels in the wildtype, PHD2, OGDH and LIAS and KO HeLa clones displayed as a heat map. Two null clones for each OGDH and LIAS KO were used and five independent cell cultures were measured for each KO clone.

Figure S5: Bioenergetic profiling and Mitochondrial ROS production in HeLa cells depleted for PHD2, OGDH and LIAS (related to Figure 4).

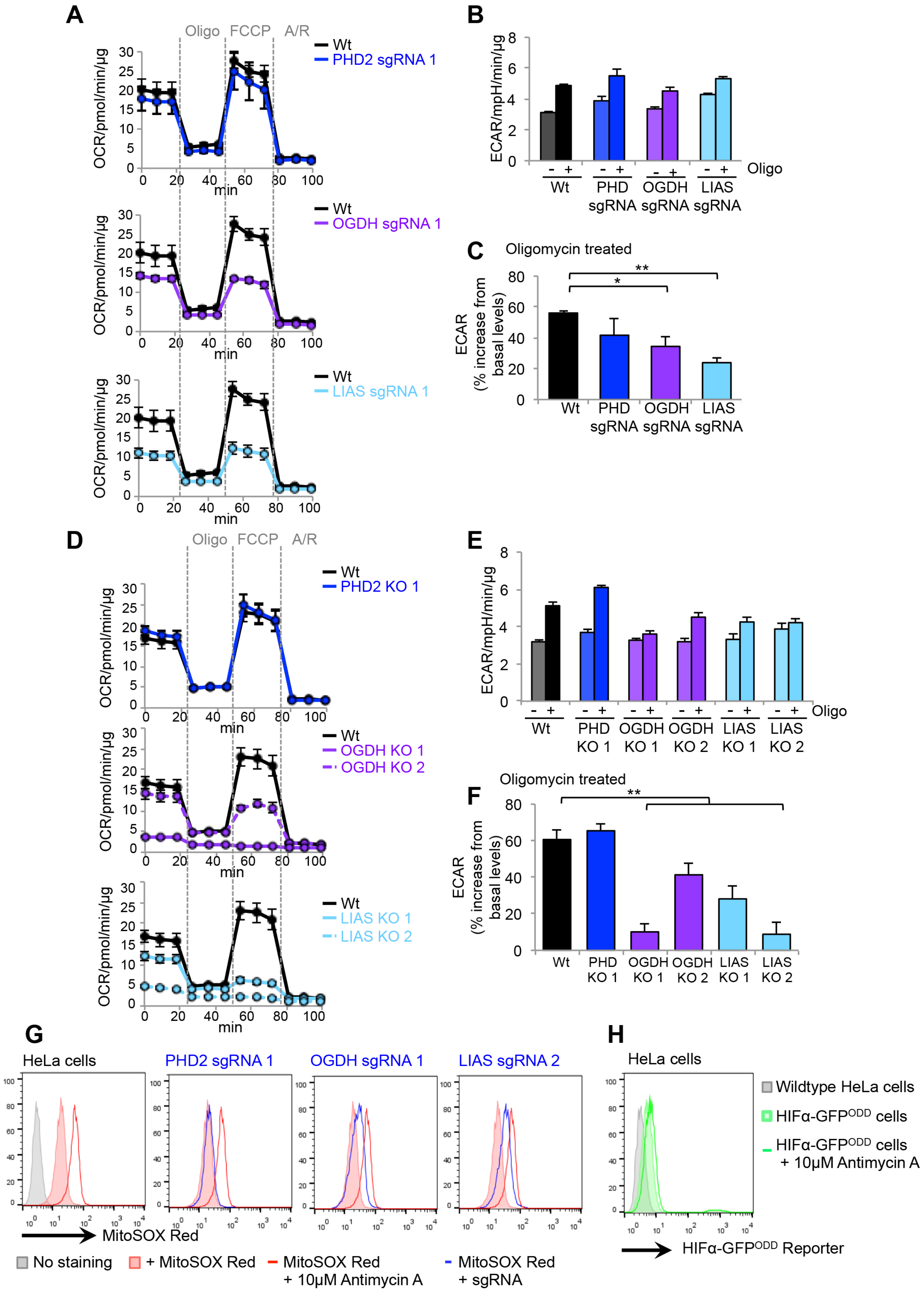

A-C, Oxygen consumption rates (OCR) (**A**) and extracellular acidification rates (ECAR) (**B, C**) were measured in HeLa cells depleted for PHD2, OGDH or LIAS. Measurements were performed using a Seahorse XF^{e}24 Extracellular Flux Analyzer (4 technical replicates per sample). Three basal measurements were made at 9 min intervals followed by three measurements per treatment (1μM oligomycin, 1μM FCCP and 1μM antimycin/rotenone). OCR and ECAR were normalised to total cell protein content. ECAR were measured at baseline and following 1μM oligomycin treatment (**B**) and the percentage increase in ECAR following oligomycin measured (**C**). **D-F** OCR (**D**) and ECAR (**E, F**) measured in the PHD2, OGDH and LIAS null clones as described previously (n=3). **G,** HeLa cells were transduced with lentivirus encoding Cas9 and sgRNAs to PHD2, OGDH or LIAS as described previously. After 10 days the cells were stained with MitoSOX Red (Thermo Fisher) and mitochondrial ROS measured by flow cytometry. Treatment with 10μM Antimycin A for 30 minutes was used as a positive control to generate mitochondrial ROS by inhibiting the oxidation of ubiquinone. **H,** HIF1α-GFP^{ODD} cells were treated with 10μM Antimycin A for 12 hours and GFP levels measured by flow cytometry. Mitochondrial ROS generated by treatment with Antimycin A did not stabilise the HIF1α-GFP^{ODD} reporter. Values are mean±SEM. *$P<0.05$, **$P<0.01$.

Figure S6: Disruption of the OGHC leads to decreased PHD and TET activity through a 2-OG dependent increase in L-2-HG (related to Figures 5 and 6).

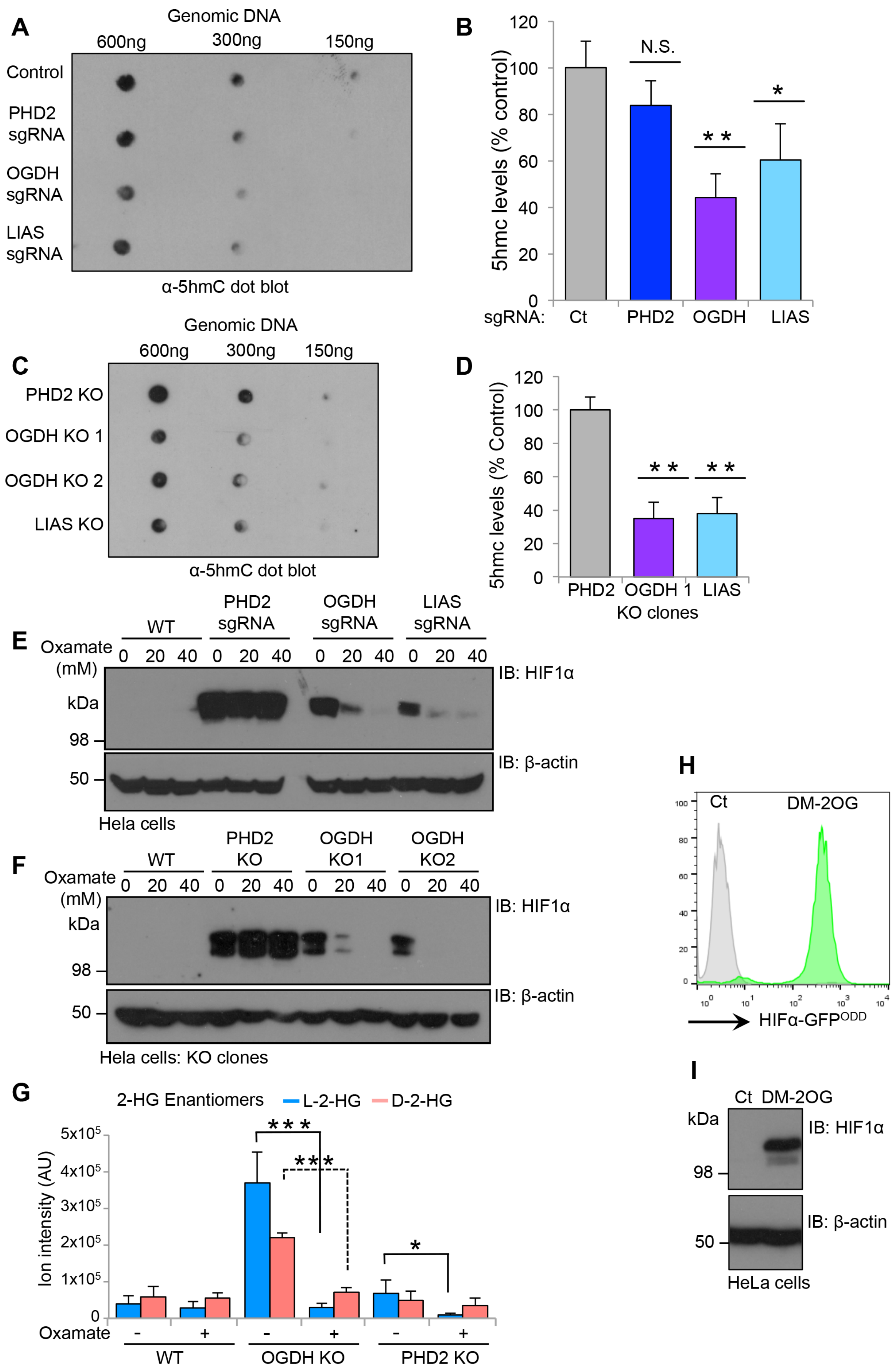

A, B, TET enzyme activity in HeLa mixed CRISPR populations depleted of PHD2, OGDH or LIAS populations. Total genomic DNA levels were measured by methylene blue staining, and the relative levels of 5-hmC quantified by densitometry (**B**) n=3. **C, D,** TET enzyme activity in PHD2, OGDH or LIAS null clones measured by probing total genomic DNA for 5-hmC. Total genomic DNA levels were measured by methylene blue staining, and the relative levels of 5-hmC quantified by densitometry (**D**) n=3. **E, F,** HIF1α levels in oxamate treated wildtype HeLa cells depleted for OGDH, LIAS or PHD2 by sgRNA (**E**) or null clones (**F**) as described**. G,** Levels of 2-HG enantiomers in wildtype, PHD2 or OGDH KO cells following 24 h oxamate treatment (40mM). Chiral derivatisation was used to label L-2-HG and D-2-HG for analysis by LC-MS. Enantiomer levels were measured by ion intensity. n=5. **H, I** HIF1α-GFP^{ODD} reporter or wildtype HeLa cells were treated with 4mM DM2-OG for 24 h and HIF1α levels visualised by GFP levels (**H**) or immunoblot (**I**). Values are mean±SEM. *P<0.05. **P<0.01. ***P<0.001 comparing + or – oxamate. N.S.= not significant

Figure S7: Human germline mutations in lipoic acid synthesis stabilise HIF1α (related to Figure 7).

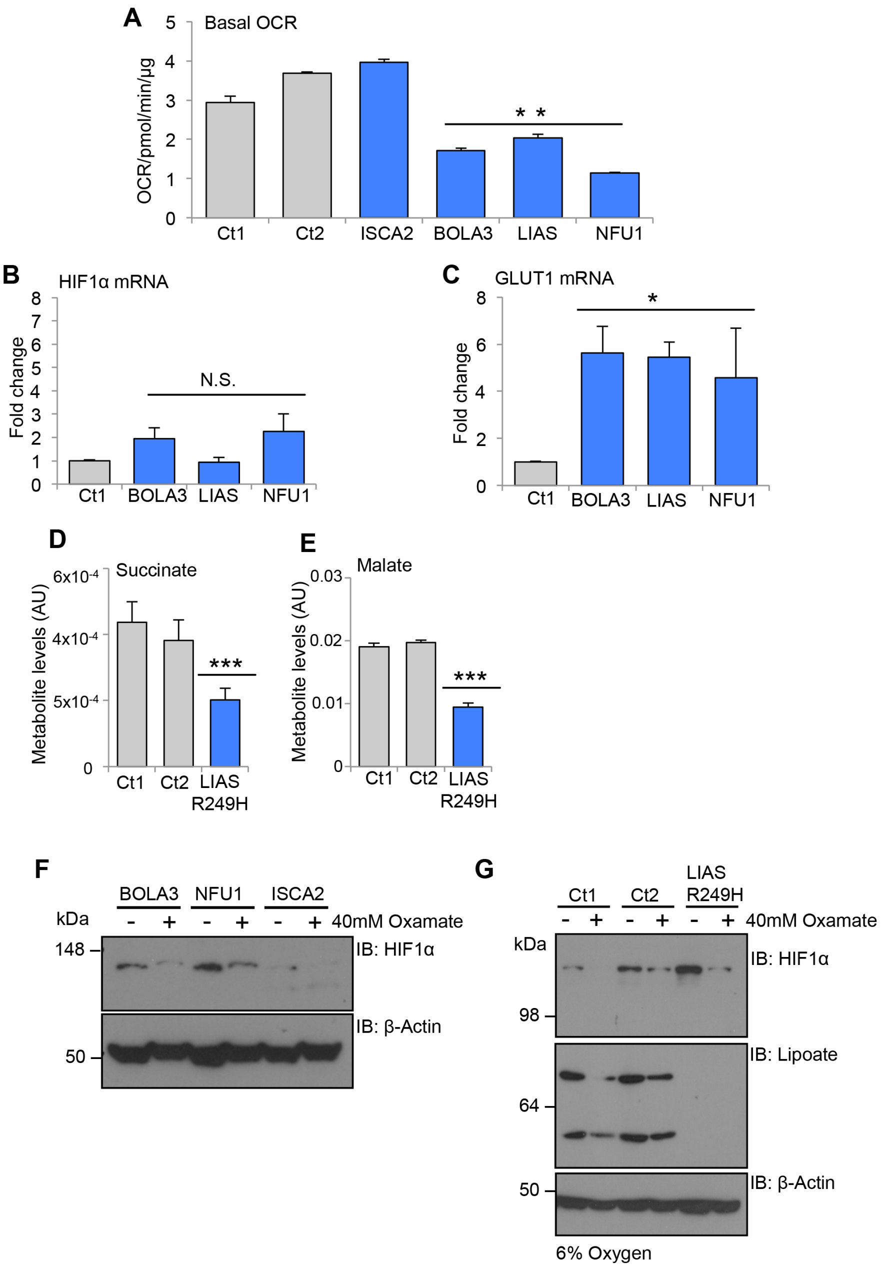

A, Basal OCR in patient control and mutant (ISCA2, BOLA3, LIAS and NFU1) fibroblast cells. **B, C,** mRNA levels of HIF1α (**B**) and GLUT1 (**C**) in control fibroblasts or fibroblast from patients with mutations in LIAS or BOLA3 were analysed by qPCR. n=3. **D, E,** Relative intracellular abundance of succinate (**D**) and malate (**E**), in control and LIAS R249H mutant fibroblasts measured by LC-MS as described. **F,** HIF1α levels following oxamate treatment of fibroblasts with homozygous mutations in the Fe-S cluster synthesis genes BOLA3, NFU1 and ISCA2. **G,** Oxamate treatment on control and LIAS mutant fibroblasts in 6% oxygen. Control (Ct) or LIAS R249H fibroblasts were incubated at 6% oxygen and treated with 40mM oxamate for 24 h. HIF1α and lipoate levels were measured by immunoblot. β-actin served as a loading control. Fibroblasts from two unrelated patients were used as controls. Values are mean±SEM. *P<0.05, **P<0.01.

Table S1: Intracellular concentrations of 2-HG (related to Figures 5-7).

sample	2-HG peak area	extract (umoles/mL)	umoles 2-HG/cell	2-HG/cell volume (uM)	Mean	SD
**HeLa control 1**	5.46E+07	7.46E-04	7.46E-10	169		
**HeLa control 2**	5.44E+07	7.44E-04	7.44E-10	168		
**HeLa control 3**	6.34E+07	8.34E-04	8.34E-10	189		
**HeLa control 4**	5.06E+07	7.06E-04	7.06E-10	160		
**HeLa control 5**	4.76E+07	6.76E-04	6.76E-10	153	**168**	**13**
**OGDH KO 1**	3.07E+08	3.27E-03	3.27E-09	651		
**OGDH KO 2**	3.19E+08	3.39E-03	3.39E-09	676		
**OGDH KO 3**	3.29E+08	3.49E-03	3.49E-09	696		
**OGDH KO 4**	3.02E+08	3.22E-03	3.22E-09	642		
**OGDH KO 5**	3.28E+08	3.48E-03	3.48E-09	694	**672**	**24**
**HeLa DM2-OG 1**	1.79E+08	1.99E-03	1.99E-09	449		
**HeLa DM2-OG 2**	4.98E+08	5.18E-03	5.18E-09	1173		
**HeLa DM2-OG 3**	5.42E+08	5.62E-03	5.62E-09	1272		
**HeLa DM2-OG 4**	4.59E+08	4.79E-03	4.79E-09	1084		
**HeLa DM2-OG 5**	3.88E+08	4.08E-03	4.08E-09	922	**980**	**323**

Table showing the mass spectrometry quantification of 2-HG performed by interpolation of the corresponding standard curve obtained from serial dilutions of DL-alpha-hydroxyglutaric acid disodium salt. Five replicates per experimental condition were performed. Cell volumes were measured by to calculate the final 2-HG concentrations (mean±SD).

EXPERIMENTAL PROCEDURES

Metabolomic analyses

Liquid Chromatography Mass Spectrometry (LC-MS) analysis of sample extracts was performed on a QExactive Orbitrap mass spectrometer coupled to Dionex UltiMate 3000 Rapid Separation LC system (Thermo). The liquid chromatography system was fitted with a SeQuant Zic-pHILIC (150mm × 2.1mm, 5μm) with guard column (20mm × 2.1mm, 5μm) from Merck (Darmstadt, Germany). The mobile phase was composed of 20mM ammonium carbonate and 0.1% ammonium hydroxide in water (solvent A), and acetonitrile (solvent B). The flow rate was set at 180μL x min^{-1} with the following gradient: 0 min 70% B, 1 min 70% B, 16 min 38% B, 16.5 min 70% B, hold at 70% B for 8.5 min. The mass spectrometer was operated in full MS and polarity switching mode. Five independent cell cultures were measured for each condition and samples were randomised in order to avoid bias in sample analyses due to machine drift. The acquired spectra were analysed using XCalibur Qual Browser and XCalibur Quan Browser software (Thermo Scientific) by referencing to an internal library of compounds. R packages muma and gplots were used for data visualisation.

Quantification of 2-HG performed by interpolation of the corresponding standard curve obtained from serial dilutions of DL-alpha-hydroxyglutaric acid disodium salt (Sigma Aldrich) running with the same batch of samples. Mean cell volume of wildtype and OGDH KO HeLa cells was measured using a CASY cell analyser. Three separate measurements were made for each cell line and averaged to give a final mean cell volume.

Derivatisation of 2-HG metabolite extracts with diacetyl-L-tartaric anhydride (DATAN) was performed as previously described(Oldham et al., 2015; Struys et al., 2004) with some modifications. Briefly, for each sample, 200μl metabolite extract was evaporated to dryness at ambient temperature in a Savant SpeedVac Concentrator (Thermo Scientific). The residue was resuspended in 50μl freshly-mixed 80% acetonitrile/20% acetic acid plus 50mg/ml DATAN (Acros Organics) and heated at 75°C for 30 min. Samples were cooled to room temperature and centrifuged before addition of 50μl 80% acetonitrile/20% acetic acid.

5μL of derivatised sample was injected onto am Acquity UHPLC HSS T3 column (100 x 2.1mm, 1.8μm particle size). A Dionex UltiMate 3000 Rapid Separation LC was coupled to a Q Exactive Orbitrap mass spectrometer, and a gradient elution of 1.5mM ammonium formate in water (mobile phase A, pH 3.6 adjusted with formic acid) and 0.1% formic acid in acetonitrile (mobile phase B) was used to separate the derivatised L- and D-2HG enantiomers. The target analytes were monitored by in parallel-reaction monitoring (PRM) in negative electrospray ionization mode. The transition (precursor ion → product ion) of m/z 363 → 147 for

derivatised D- and L-2-HG was monitored and the accurate mass of 147.03 was extracted using XCalibur Qual Browser and XCalibur Quan Browser software (Thermo Scientific), and used for relative quantification. Experimental samples were randomised in order to avoid bias in sample analyses due to machine drift.

Modelling of the OGDHC and lipoic acid synthesis proteins

All non-experimentally derived models were determined through use of the Phyre2 fold recognition server(Kelley et al., 2015). The E1 subunit, OGDH (Uniprot id Q02218) was submitted to the Phyre2 server and a model was returned matching residues 36-975 (95% coverage), based on *mycobacterium smegmatis* alpha-ketoglutarate decarboxylase (PDB id 2XT6, 44% sequence identity). The E2 subunit, DLST (Uniprot id P36957), was modelled by considering each of its domains separately and submitting them to the Phyre2 server. Residues 220-451, corresponding to the catalytic domain, were modelled based on the *E. coli* DLST catalytic domain (PDB id 1SCZ, 60% sequence identity). Residues 71-143, corresponding to the lipoyl domain, were modelled on the lipoyl domain of PDH kinase isoform 3 (PDB id 2Q8I, 40% identity). As the OGDHC shares its E3 subunit, DLD, with PDH, a crystal structure of this subunit, in the form of a dimeric biological assembly, was obtained from the PDB (id 1ZMD) and used directly to generate an image. Figures derived from these models were prepared by generating space-filling sphere based images using PyMOL.

Proteins involved in lipoic acid formation were modelled as for the OGDHC, and were generated via the Phyre2 server. ISCA2 (Uniprot id Q86U28) residues 49-153 were modelled on IscA from *thermosynechococcus elongatus* (PDB id 1X0G, 27% identity). The location of the [2Fe-2S] cluster was copied from that in the template structure by structural alignment using the super PyMOL command. BOLA3 (Uniprot id Q53S33) residues 33-107 were modelled on BolA2 from *arabadopsis thaliana* (PDB id 2MM9). An NMR model (PDB id 2M5O) exists for residues 162-247 of NFU1 and was used directly in visualisation. LIAS residues 70-357 were modelled on lipoyl synthase 2 from *thermosynechococcus elongatus* (PDB id 4U0O). To place the [4Fe-4S] clusters in the human LIAS model, the template and model were aligned to each other with the PyMOL align command and clusters copied to the human model.

Bioenergetic Profiling

OCR and ECAR measurements were performed using a Seahorse XF^{e}24 Extracellular Flux Analyzer. HeLa cells were seeded to Seahorse XF^{e}24 cell culture microplates and 1 h prior to analysis the medium was replaced with unbuffered DMEM (25mM glucose, 2mM L-glutamine, 1mM sodium pyruvate, 2% FCS). Measurements were performed as described by

Nicholls et al(Nicholls et al., 2010) with some modifications. Briefly, three basal OCR and ECAR measurements were taken at 9 min intervals, followed by sequential treatments with 1μM oligomycin, 1μM FCCP and 1μM rotenone/1μM antimycin (three measurements each at 9 min intervals). Following analysis, the cells were lysed in 1% NP40 and the protein content of each well determined by Bradford protein assay to allow normalisation of OCR and ECAR values to cell protein content.

Patient Samples

Primary human fibroblast cell lines were kindly donated by Dr Johannes Mayr. The following cell lines have been published previously:

1) LIAS (NM_006859.2) c.746G>A (p.Arg249His), homozygous (Mayr et al., 2011).

2) BOLA3 (NM_212552.2) c.200T>A (p.Ile67Asn), homozygous (published as patient #49720) (Haack et al., 2013).

3) NFU1 (NM_001002755.2) c.[544C>T];[?] p.[Arg182Trp];[?]. This patient was investigated for an NFU1 mutation and the mutation c.544C>T was identified (published as patient 3) (Ahting et al., 2015). At the level of genomic DNA this position was found to be heterozygous. All coding exons were sequenced but a second mutation could not be identified. It is most likely the second allele results in an unstable mRNA, probably due to activation of a cryptic intronic splice site.

ISCA2 (NM_194279.3) c.G229A (p.Gly77Ser), homozygous . This patient with an ISCA2 mutation was born as the first child of healthy consanguineous parents (first degree cousins) of Arabian origin. Initially she showed normal psychomotor progress until the age of 6 months when she had a developmental arrest. She failed to thrive and dropped under the 3rd percentile of head circumference, body length and weight. She developed severe muscular hypotonia, seizures, sensorineural hearing loss, optic atrophy and blindness and lost all her skills. Chronic respiratory failure necessitated tracheostomy and artificial ventilation. She died at the age of 2 years. Laboratory evaluation showed the following significant abnormalities: increased lactate in cerebrospinal fluid (CSF) (8.3 mmol/l, normal 0,8 – 2,5 mmol/l), glycine elevatation in plasma (1039 μmol/l, normal 73-436) and CSF (45 μmol/l, normal 2-8). Alanine was elevated in CSF (90 μmol/l, normal 13-45). An MRI revealed severe leukodystrophy already at age 7 months showing progression at age 22 months. This mutation has been previously described and seems to be a founder mutation in the Saudi population (Al-Hassnan et al., 2015).

Two fibroblast control cells were used:
C1, This was obtained from a girl with HMGCL deficiency (NM_000191.2) causing abnormalities in ketone body formation (donated by Dr Mayr).
C2, Female patient obtained as a primary dermal fibroblast commercial sample from Lonza.

Plasmids

Full details of plasmids used are shown below. The majority of viral vectors were pHRSIN-based lentiviral plasmids (Demaison et al., 2002). The HIF1α-GFP^{ODD} reporter was generated by insertion of the HIF1α ODD (aa 530-603) at the 5' of EGFP in the pHRSIN EGFP vector. The SFFV promoter was substituted for the iNOS HRE minimal promotor region to form the pHRSIN HRE- HIF1α-GFP^{ODD} plasmid. The LentiCRISPRv2 vector system was used for all CRISPR-Cas9 experiments (Sanjana et al., 2014).

Name	Transgene	Source
pHRSIN-pSFFV-EGFP-pGK-Puro	EGFP	P. Lehner (Cambridge)
Z-loxP-mCherry	mCherry gene-trap	P. Lehner (Cambridge)
pCMVR8.91	Lentiviral gag/pol	P. Lehner (Cambridge)
pMD.G	Lentiviral VSVG	P. Lehner (Cambridge)
pMD.GagPol	Retroviral gag/pol	P. Lehner (Cambridge)
pMD.VSVG	Retroviral VSVG	P. Lehner (Cambridge)
pCDNA3 HIF1αHA	HIF1α	P. Maxwell (Cambridge)
pGL-HRE	iNOS HRE	S. Rocha (Dundee)
OGDH IMAGE Clone	OGDH	Source Bioscience
LIAS IMAGE Clone	LIAS	Source Bioscience
LentiCRISPR v2	sgRNA/CAS9	F. Zhang (Addgene #52961)
gBlocks Gene Fragment	L2HGDH	IDT
gBlocks Gene Fragment	D2HGDH	IDT

Antibodies and Reagents

Primary Antibodies

**Protein**	**Cat no.**	**Company/Source**	**Species**
HIF1α	610959	BD Biosciences	Mouse
Hydroxy-HIF1α	#3434	Cell Signaling	Rabbit
PHD2 (EGLN1)	NB100-137SS	Novus Biologicals	Rabbit
OGDH	HPA020347	Atlas	Rabbit
Lipoate	437695	Calbiochem	Rabbit
β-Actin	A228	Sigma	Mouse
VHL	#2738	Cell Signaling	Rabbit
LDHA	#2012	Cell Signaling	Rabbit
MHC Class I (W6/32)		P. Lehner (Cambridge)	Mouse
β2 microglobulin	A00072	Dako	Rabbit
CA9 (M75)		E. Oosterwijk (Radboud)	Mouse
Malate dehydrogenase 1	NBP1-89515	Novus Biologicals	Rabbit
Malate dehydrogenase 2	AB96193	Abcam	Rabbit
L2HGDH	15707-1-AP	Proteintech	Rabbit
D2HGDH	13895-1-AP	Proteintech	Rabbit
5-Hydroxymethylcytosine	39770	Active Motif	Rabbit
Goat Anti-rabbit	HRP	111-035-045	Jackson
Goat Anti-mouse	AF488	A11029	Life Tech
Goat Anti-rabbit	AF488	A11034	Life Tech
Goat Anti-mouse	AF568	A11031	Life Tech
Goat Anti-rabbit	AF568	A11036	Life Tech
Goat Anti-mouse	AF647	A21236	Life Tech
Goat Anti-rabbit	AF647	A21245	Life Tech

CRISPR sgRNAs

**Target Gene**	**Guide no.**	**sgRNA sequence**
PHD2	1	ATGCCGTGCTTGTTCATGCA
VHL	1	GCCGTCGAAGTTGAGCCATA
β2 microglobulin	1	GGCCGAGATGTCTCGCTCCG
OGDH	1	CTGCTCTTACCTCCAGCCGA
	2	TTCCTGTCCCCCGATGAAAG
LIAS	1	TTAGGTTAAGACTACCTCCA
	2	GAAGCTCGATGTCCCAATAT
DLST	1	AGCCTACCAGGTTTGCCAGA
	2	TGCAGGGGTCTCCTTATGCC
	3	TACACAACCTTCCTGCTGTT
DLD	1	TGATCTGCGTAAGTTCTCAG
	2	ATAGCAGCAACATATCCTCC
	3	TGTGGTAGTATCTATGCCAT

siRNAs

Target Gene	Oligo no.	siRNA sequence
LDHA	1	GGAGAAAGCCGUCUUAAUU
	2	GGCAAAGACUAUAAUGUAA
	3	UAAGGGUCUUUACGGAAUA
	4	AAAGUCUUCUGAUGUCAUA
MDH1	1	CCUUAGAUAAAUACGCCAA
	2	GGGAGAAUUUGUCACGACU
	3	CAACAGAUAAAGAAGACGU
	4	AGGUUAUUGUUGUGGGUAA
MDH2	1	GGGUUUGGAUCCAGCUCGA
	2	GAUCUGAGCCACAUCGAGA
	3	CGGAGGUGGUCAAGGCUAA
	4	CGCCUGACCCUCUAUGAUA

Reagent	Cat No.	Company
(D)-2-Hydroxyglutarate	H8378	Sigma-Aldrich
(L)-2-Hydroxyglutarate	90790	Sigma-Aldrich
13C5 L-glutamine	CLM-1822-H-MPT	Cambridge Isotope Laboratories
Accuprime Taq	12339-016	Life Tech
Agencourt AMPure XP beads	A63880	Beckman Coulter
Antimycin	A8674	Sigma-Aldrich
DATAN	6283-74-5	Acros Organics
α-ketoglutaric acid	K1128	Sigma-Aldrich
DMOG	D3695	Sigma-Aldrich
FCCP	sc-203578	Santa Cruz
HEPES	H0887	Sigma-Aldrich
M280 Streptavidin Dynabeads	11205D	Thermo Fisher
MitoSOX Red	M36008	Thermo Fisher
Oligofectamine transfection reagent	12252011	Thermo Fisher
Oligomycin	75351	Sigma-Aldrich
Polybrene	H9268	Sigma-Aldrich
Prolong-Gold Antifade with DAPI	#8961	Cell Signalling
Propidium Iodide	P4864	Sigma-Aldrich
Rotenone	R8875	Sigma-Aldrich
Sodium L-Lactate	71718	Sigma-Aldrich
Sodium Oxamate	O2751	Sigma-Aldrich
Trans-IT 293 reagent	MIR 2700	Mirus
VEGF DuoSet ELISA	DY293B	R&D Systems

References:

Ahting, U., Mayr, J.A., Vanlander, A.V., Hardy, S.A., Santra, S., Makowski, C., Alston, C.L., Zimmermann, F.A., Abela, L., Plecko, B., et al. (2015). Clinical, biochemical, and genetic spectrum of seven patients with NFU1 deficiency. Frontiers in genetics *6*, 123.

Al-Hassnan, Z.N., Al-Dosary, M., Alfadhel, M., Faqeih, E.A., Alsagob, M., Kenana, R., Almass, R., Al-Harazi, O.S., Al-Hindi, H., Malibari, O.I., et al. (2015). ISCA2 mutation causes infantile neurodegenerative mitochondrial disorder. Journal of medical genetics *52*, 186-194.

Demaison, C., Parsley, K., Brouns, G., Scherr, M., Battmer, K., Kinnon, C., Grez, M., and Thrasher, A.J. (2002). High-level transduction and gene expression in hematopoietic repopulating cells using a human immunodeficiency [correction of imunodeficiency] virus type 1-based lentiviral vector containing an internal spleen focus forming virus promoter. Human gene therapy *13*, 803-813.

Haack, T.B., Rolinski, B., Haberberger, B., Zimmermann, F., Schum, J., Strecker, V., Graf, E., Athing, U., Hoppen, T., Wittig, I., et al. (2013). Homozygous missense mutation in BOLA3 causes multiple mitochondrial dysfunctions syndrome in two siblings. Journal of inherited metabolic disease *36*, 55-62.

Kelley, L.A., Mezulis, S., Yates, C.M., Wass, M.N., and Sternberg, M.J. (2015). The Phyre2 web portal for protein modeling, prediction and analysis. Nature protocols *10*, 845-858.

Mayr, J.A., Zimmermann, F.A., Fauth, C., Bergheim, C., Meierhofer, D., Radmayr, D., Zschocke, J., Koch, J., and Sperl, W. (2011). Lipoic acid synthetase deficiency causes neonatal-onset epilepsy, defective mitochondrial energy metabolism, and glycine elevation. American journal of human genetics *89*, 792-797.

Nicholls, D.G., Darley-Usmar, V.M., Wu, M., Jensen, P.B., Rogers, G.W., and Ferrick, D.A. (2010). Bioenergetic profile experiment using C2C12 myoblast cells. Journal of visualized experiments : JoVE.

Oldham, W.M., Clish, C.B., Yang, Y., and Loscalzo, J. (2015). Hypoxia-Mediated Increases in L-2-hydroxyglutarate Coordinate the Metabolic Response to Reductive Stress. Cell metabolism *22*, 291-303.

Sanjana, N.E., Shalem, O., and Zhang, F. (2014). Improved vectors and genome-wide libraries for CRISPR screening. Nature methods *11*, 783-784.

Struys, E.A., Jansen, E.E., Verhoeven, N.M., and Jakobs, C. (2004). Measurement of urinary D- and L-2-hydroxyglutarate enantiomers by stable-isotope-dilution liquid chromatography-tandem mass spectrometry after derivatization with diacetyl-L-tartaric anhydride. Clin Chem *50*, 1391-1395.
